# Safety and efficacy of intravenous tenecteplase in patients with acute ischemic stroke in extended time window: systematic review and meta-analysis

**DOI:** 10.1186/s40001-025-03466-7

**Published:** 2025-11-29

**Authors:** Abdelmonam M. Hagag, Gannah S. El Labban, Abdelrhman M. Asar, Omar R. Ayad, Salma Adel Ezzat, Mostafa A. Fathi, Mohamed Mohsen Helal

**Affiliations:** 1 Faculty of Medicine, Zagazig University, Zagazig, Egypt; 2 Faculty of Medicine, Ain Shams University, Cairo, Egypt; 3 Mansoura Manchester Program for Medical Education, Faculty of Medicine MBBCh, Mansoura University, Mansoura, Egypt; 4 Faculty of Medicine, Kafr Elsheikh University, Kafr Elsheikh, Egypt

**Keywords:** Tenecteplase, TNK, Acute ischemic stroke, AIS, Extended time window

## Abstract

**Background:**

There is limited evidence regarding the safety and efficacy of tenecteplase 0.25 mg/kg in patients with acute ischemic stroke in the extended time window, either with large vessel occlusion or not. Therefore, we aim to assess its safety and efficacy for these patients.

**Methodology:**

We searched PubMed, Scopus, and Web of Science for randomized controlled trials that compared tenecteplase 0.25 mg/kg with the best medical management. Our primary efficacy outcomes included favorable and excellent functional outcomes, measured via the modified Rankin score (mRS), and early neurological improvements. Primary safety outcomes were mortality and symptomatic intracranial hemorrhage rates. Subgroup analyses were performed based on mRs, and the inclusion of patients eligible for mechanical thrombectomy in addition to TNK.

**Results:**

Our search found nine randomized controlled trials, of which 8 were included in the meta-analysis. A total of 3068 patients were included. Tenecteplase 0.25 mg/kg did not differ from the control group in achieving excellent functional outcomes and favorable functional outcomes (*P* values: 0.15 and 0.69), while there was significant improvement in early neurological improvement (*P* value: 0.02). Symptomatic intracranial hemorrhage was statistically higher in the tenecteplase 0.25 mg/kg group (*P* value 0.008). Subgroup analyses indicated that patients with mRS 0–1 who receive TNK 0.25 mg/kg do better than those with mRS 0–2. Better results for TNK 0.25 mg/kg were observed in studies that excluded patients eligible for mechanical thrombectomy in addition to TNK.

**Conclusions:**

TNK 0.25 mg/kg appears to significantly achieve early neurological improvement with no effect in achieving excellent or favorable functional outcomes; however, it showed a significant increase in symptomatic intracranial hemorrhage. Patients who performed endovascular thrombectomy after administering TNK 0.25 mg/kg had statistically significantly higher rates of symptomatic intracranial hemorrhage; on the other hand, current evidence endorses the use of tenecteplase 0.25 mg/kg in patients with ischemic strokes whose baseline mRs ranges from 0 to 1. Further RCTs are necessary to validate these findings. *Prospero registration number*: CRD42025636473.

**Supplementary Information:**

The online version contains supplementary material available at 10.1186/s40001-025-03466-7.

## Introduction

With the aging of the global population and the increasing prevalence of cardiovascular diseases, the burden of ischemic stroke has risen significantly. From 1990 to 2019, ischemic stroke prevalence increased by 102%, making it the second leading cause of death worldwide [1, 2]. According to the latest report from the World Stroke Organization (WSO), ischemic stroke imposes a substantial economic burden, costing the global economy approximately US$721 billion annually [1].

The primary goal in treating acute ischemic stroke (AIS) is achieving timely reperfusion, either through intravenous thrombolysis or, when necessary, endovascular interventions. However, time is a critical factor in AIS management, as patient outcomes are highly dependent on the rapid restoration of blood flow [3]. Over the years, significant research efforts have focused on extending the time window for reperfusion eligibility. Identifying new solutions or testing alternative medications for this purpose remains a pressing need.

For patients with stroke onset within 4.5 h, intravenous tissue plasminogen activator (alteplase) has long been the standard of care [4]. Previous studies have shown that tenecteplase is non-inferior to alteplase [5, 6], and there is recent evidence that shows it may even be superior [7, 8]. This evidence has prompted updates in early clinical guidelines in the United States, Europe, Canada, and Australia, which now recommend TNK as an alternative to alteplase [4, 9–11]. The Australian guidelines now prioritize TNK as the first-line treatment for these populations, reserving alteplase only if TNK is unavailable [11]. Compared to alteplase, TNK offers several pharmacological advantages, including greater fibrin specificity (resulting in less systemic depletion of circulating fibrinogen) and 80-fold greater resistance to plasminogen activator inhibitors. The latter allows for a longer half-life, enabling TNK to be administered as a single intravenous bolus rather than the continuous infusion required for alteplase [12, 13].

Endovascular thrombectomy is another therapy that has shown efficacy in patients with AIS, specifically in those with large vessel occlusion [4]. There is significant evidence that EVT is effective at improving outcomes in the extended time window [14]. However, the efficacy of thrombectomy is lower in patients who present later than in those treated earlier [15]. This may be partly due to the low percentage of patients receiving thrombolytic therapy beyond 4.5 h [16]. Emerging evidence suggests that TNK may offer advantages in this context. A meta-analysis of ten studies demonstrated that TNK, when used in conjunction with mechanical thrombectomy within 4.5 h of onset, achieved higher rates of early recanalization without increasing the risk of intracerebral hemorrhage, and lower rates of 90-day mortality than alteplase did [17].

The utility of thrombolytic therapy beyond 4.5 h remains a topic of debate. Interestingly, a systematic review of nine randomized trials comparing alteplase to placebo or open controls across different time windows (< 3 h, 3–4.5 h, and > 4.5 h) revealed that alteplase administered beyond 4.5 h provided the least therapeutic benefit, with no significant treatment effect observed in this window. In addition, alteplase was associated with a significant increase in symptomatic and fatal intracranial hemorrhage, with the risk of fatal hemorrhage remaining consistent across treatment delays, ages, and stroke severities [18]. New studies have emerged after this study with different results. The WAKE-UP [19] trial was an RCT published in 2018 that investigated the effect of alteplase in patients with stroke of unknown onset. They showed that alteplase was superior to placebo in achieving favorable and excellent functional outcomes, while no difference was observed in the safety measures, such as death and symptomatic intracranial hemorrhage. The EXTEND trial [20] that was published in 2019 showed similar results, as placebo achieved a statistically significant improvement in excellent functional outcomes compared to placebo, with no statistically significant increase in death or intracranial hemorrhage.

On the other hand, the unique pharmacological properties of TNK are hypothesized to enable faster reperfusion and lower rates of intracranial hemorrhage [21, 22]. Palaiodimou et al. [7] was a meta-analysis that assessed the safety and efficacy of TNK in extended-window stroke patients. They included three RCTs, demonstrating favorable outcomes with an increase in symptomatic intracranial hemorrhage in the TNK group; however, their conclusions are limited due to small sample sizes [23].

This systematic review and meta-analysis aimed to consolidate existing data on TNK’s efficacy and safety beyond 4.5 h post-stroke onset. We seek to inform clinical guidelines and refine therapeutic approaches for this underserved population.

## Methodology

This systematic review was registered a priori on the International Prospective Register of Systematic Reviews PROSPERO (Registration No: CRD42025636473). All steps in the review process were conducted according to the methodological guidelines reported in the Cochrane Handbook for Systematic Reviews of Interventional Studies [24]. We followed the Preferred Reporting Items for Systematic Reviews and Meta-Analysis (PRISMA) guidelines in reporting the methodology and results of this systematic review and meta-analysis [25].

### Eligibility criteria

We considered only studies with a randomized controlled trial design, involving patients with AIS with an extended time window from 4.5 to 24 h post-stroke onset or from the last time known well. The intervention was TNK 0.25 mg/kg, and the comparator was suitable for medical management for such a condition or placebo. At least one efficacy outcome and one from the safety outcome were reported. We limited the inclusion to English-language studies only and studies involving human patients. No restrictions on publication dates were applied during the search.

We excluded non-English studies, nonrandomized studies, including observational studies, conference abstracts or protocols, and studies that involved animals.

### Search methods for the identification of studies

A comprehensive search of electronic databases, including MEDLINE via PubMed, Scopus, and Web of Science, was conducted. The search strategy was performed using the following keywords: (Ischemic stroke OR transient ischemic attack) AND (Tenecteplase). The full search strategy is provided in the supplementary material.

### Selection of studies

The results of the literature search were uploaded to the Rayyan software for screening. Four authors (A.M.A, G.S.L, S.A, and O.R.A) independently screened studies in two phases: first, abstracts were screened for eligibility, followed by full-text screening of eligible abstracts. Disagreements were resolved through discussion with another reviewer (A.M.).

### Data extraction

Four authors (G.S.L, S.A, O.R.A, and M.A.F) independently extracted data into a customized spreadsheet, captured study characteristics, and reported outcomes. Discrepancies were resolved through discussion with another investigator (A.M.). No automation or semi-automation tools or software were utilized during the data extraction process.

### Assessment of risk of bias

We utilized the Cochrane Risk of Bias 2.0 tool (RoB 2) to assess bias from an intention-to-treat perspective across five domains: randomization process, deviations from intended interventions, missing outcome data, outcome measurement, and selective reporting [26].

### Study outcomes

Our primary efficacy outcomes were favorable functional outcomes, excellent functional outcomes, and early neurological improvements.

A favorable functional outcome was defined as a modified Rankin score [27] of 0–2 at 90 days (mRS 0–2 at 90 days), while an excellent functional outcome was defined as mRS 0–1 at 90 days. On the other hand, we used the definitions for early neurological improvements in the included studies. Most studies define it as a reduction of ≥ 8 points on the NIHSS score (National Institute of Health Stroke Scale) from the baseline NIHSS score, or a return to 0–1 point at 24 h. However, some studies define it as a reduction of ≥ 4 points on the NIHSS score from the baseline at 24 h.

The primary safety outcomes were the all-cause mortality rate at 90 days and symptomatic intracranial hemorrhage (sICH). Again, we used the included articles’ definitions of sICH. Most studies used the European Cooperative Acute Stroke Study III criteria (ECASS III) definition It was defined as hemorrhage with neurological deterioration (defined by a reduction of the NIHSS score of at least 4 points or more) or death with hemorrhage being the predominant cause of death [28], also some studies used other definition based on ECASSII [29] and Safe Implementation of Treatment of Stroke (SITS) definition [30].

The secondary efficacy outcomes were the distribution of mRs at 90 days, reperfusion, recanalization, and poor functional outcomes at 90 days. Recanalization was defined based on the Arterial Occlusive Lesion scale (A scale that ranges from 0, which indicates no recanalization, to 3, which indicates complete recanalization). Reperfusion was defined based on the modified Treatment in Cerebral Infarction Scale [31] (TICI). Again, this scale ranges from 0 to 3, where 0 indicates no reperfusion and 3 indicates complete reperfusion [32]. Poor functional outcomes were defined as mRS 5–6.

The secondary safety outcomes were any intracranial hemorrhage and parenchymal hematoma type 2 (PH-2).

### Data synthesis

We performed a pairwise meta-analysis using RevMan 5.4. Relative risk with 95% CI was used, assuming that *P* value of < 0.05 is statistically significant. A random-effects model by DerSimonian and Laird [33] was employed in the meta-analysis, weighting studies via Mantel–Haenszel. We assessed the heterogeneity via *I*^2^ and Cochrane Q. A Cochrane Q *P* value of ≥ 0.1 was considered homogeneous, and *I*^2^ results of 25%, 50%, and 75% were considered as mild, moderate, and high levels of heterogeneity, respectively.

### Subgroup analysis

We conducted a subgroup analysis according to the baseline of mRs, and we also analyzed studies that compared TNK 0.25 mg/kg versus best medical management. Finally, a subgroup analysis was conducted according to the allowance of mechanical thrombectomy after the procedure.

### Sensitivity analysis

We performed a sensitivity analysis via a leave-one-out test by manually removing studies to assess any source of heterogeneity and to assess the significance of removing each study on the outcome significance.

### Assessment of reporting publication bias

We would perform publication bias if there were outcomes with at least 10 studies.

## Results

**Fig. 1:**
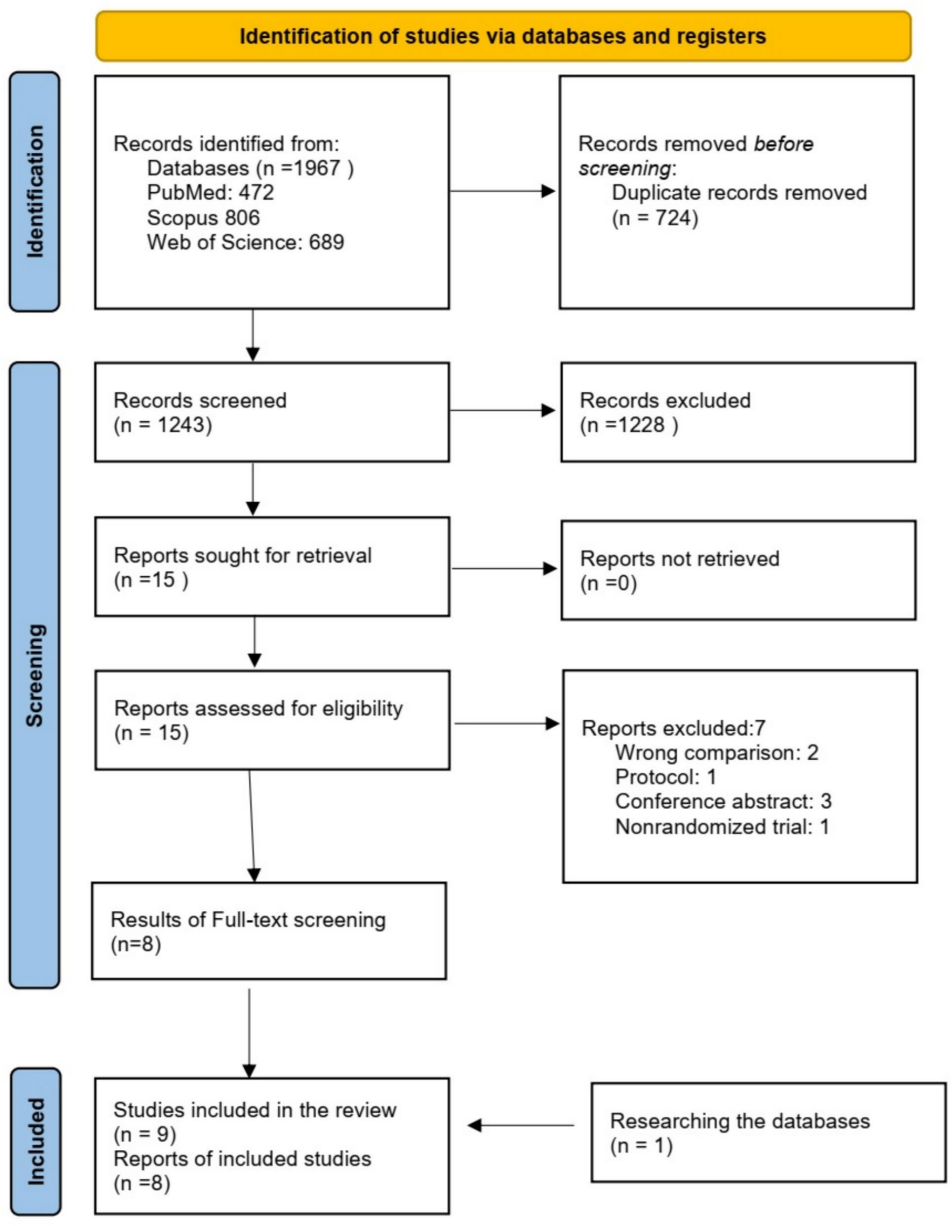
PRISMA 2020 flow chart

We searched PubMed, Scopus, and Web of Science till 1 st January 2025, revealing 1967 articles, of which 724 were excluded due to duplicates. The remaining 1243 articles were included in the title and abstract screening, resulting in 15 articles that were included in the full-text screening. Finally, we include 8 randomized controlled trial articles [15, 34–40]. We re-checked the databases upon completion on the 20th September and found a new RCT that met the inclusion criteria [41]. Therefore, the final number of the included studies was 9. Figure 1 represents the PRISMA chart that summarizes the inclusion process.

### Characteristics of the included articles

Among the included articles, 8 were open-label, blinded endpoint randomized control trials, while Albers [39] was a double-blinded randomized control trial. 6 articles compared TNK 0.25 mg/kg taken as an intravenous bolus dose versus best medical management, such as antiplatelets, and another supportive measurement according to local protocols [15, 34–36, 38], while Albers [39] compared TNK 0.25 mg/kg versus placebo. Cheng 2024 compared TNK 0.25 mg/kg as the intervention group in comparison with TNK 0.32 mg/kg as a comparison group [37], and Chen 2024 compared a combination of 0.25 mg/kg TNK and butylphthalide with butylphthalide alone [40]. Butylphthalide is a cerebroprotective agent that reduces infarct volume, brain edema, and neuronal cell death and maintains the blood–brain barrier intact [42–44]. Finally, Cheng 2024 was not included in the meta-analysis due to a different control group from other studies.

Post-procedure mechanical thrombectomy was allowed for patients in 5 studies [15, 35–37, 39] according to the status of the patients, while it was not allowed at all in 3 studies [34, 38, 40]; however, Xiong [38] excluded patients who had access to thrombectomy before the procedure, although post-procedure thrombectomy was allowed when needed. All studies included patients with stroke within 4.5–24 h of onset; however, Roaldsen 2023 included patients with stroke within 4.5 h of awakening, and Chen 2024 included patients with stroke within 4.5–6 h of onset. Table 1 summarizes the included articles.

**Table 1:** Summary sheet

Study ID	Site	Study time	Major neurological improvement	Total Sample size	TNK group*			Control group	
					No patients	Baseline NIHSS	Name	No of Patients	Baseline NIHSS
Roaldsen 2023	Ten countries: Denmark, Estonia, Finland, Latvia, Lithuania, New Zealand, Norway, Sweden, Switzerland, and the UK	From 2017 to 2021		578	288	6 [5–11]	Standard care: Thrombectomy was allowed	290	6 [5–10]
Wang 2023	Multicenter in 14 hospitals in China	From 2021 to 2022	more than a four-point decrease in NIHSS score within 24 h	80	40	7.5 [6–10.75]	standard care according to the current national guidelines for AIS	40	7 [6.00–8.75]
Albers 2024	United States and Canada	From 2019 to 2022		458	228	12 [8–17]	Placebo	230	12 [8–18]
Cheng 2024	China	From 2019 to 2021	NIHSS 0–1 or an NIHSS improvement of ≥ 8	86	43	11 [8–15]	Tenecteplase 0.32 mg/kg	43	9 [6–13]
Coutts 2024	Australia–Austria–Brazil–Canada–Finland–Ireland–New Zealand–Singapore–Spain UK	From 2015 to 2024	NIHSS of 0 at 5 days or discharge	884	432	2 [1–3]	non-thrombolytic treatment Dual antiplatelet therapy with aspirin and clopidogrel or aspirin monotherapy	452	2 [1–3]
Chen 2024**	China	From 2022 to 2022	more than 4 four-point decrease in NIHSS within 24 h	99	50	5 [4–7]	Butylphthalide alone	49	5 [4–6]
Xiong 2024	China	From 2022 to 2023	a reduction from baseline of ≥ 8 points on the NIHSS or an NIHSS score of ≤ 1	516	264	11 [7–15]	standard medical treatment	252	10 [7–14]
Cheng 2025	China	From 2021 to 2023	defined as an ≥ eight-point reduction in the NIHSS or an NIHSS score of 0–1	224	111	9 [5–14]	best medical treatment	113	9 [6–16]
Yogendrakumar 2025	Australia	From 2020 to 2024	a reduction in the 24-h NIHSS score of ≥ 8 points or a 24-h NIHSS score of 0–1	242	120	13 [7–19]	Standard medical management	122	14 [7–18]

*TNK dose in the intervention group is 0.25 mg/kg

The maximum dose is 25 mg/kg, taken as a bolus dose

Follow-up for each study is 3 months

The total population was 3068, of which 1576 were in the TNK 0.25 mg/kg group and 1591 were in the control group. The mean age for the TNK 0.25 mg/kg group was 69.57 ± 12.68 and 69.85 ± 13 for the control group. Most patients were males across the studies; most included patients with baseline NIHSS scores of more than 5, except Coutts [36] included patients with baseline NIHSS scores of 0–5. Finally, the most reported risk factors were hypertension and diabetes (see Table S1 in supplementary material).

### Quality assessment

All studies were assessed using the Risk of Bias Tool 2 (ROB 2 tool), revealing that most studies have a low risk of bias. However, both Chang 2024 and Chang 2025 had some concerns due to differences in baseline characteristics between the intervention and control groups. Chang 2024 and Yogendrakumar 2025 had some concerns due to deviation from the intended intervention. See supplementary materials.

### Study outcomes

#### Primary efficacy outcomes

**Fig. 2:**
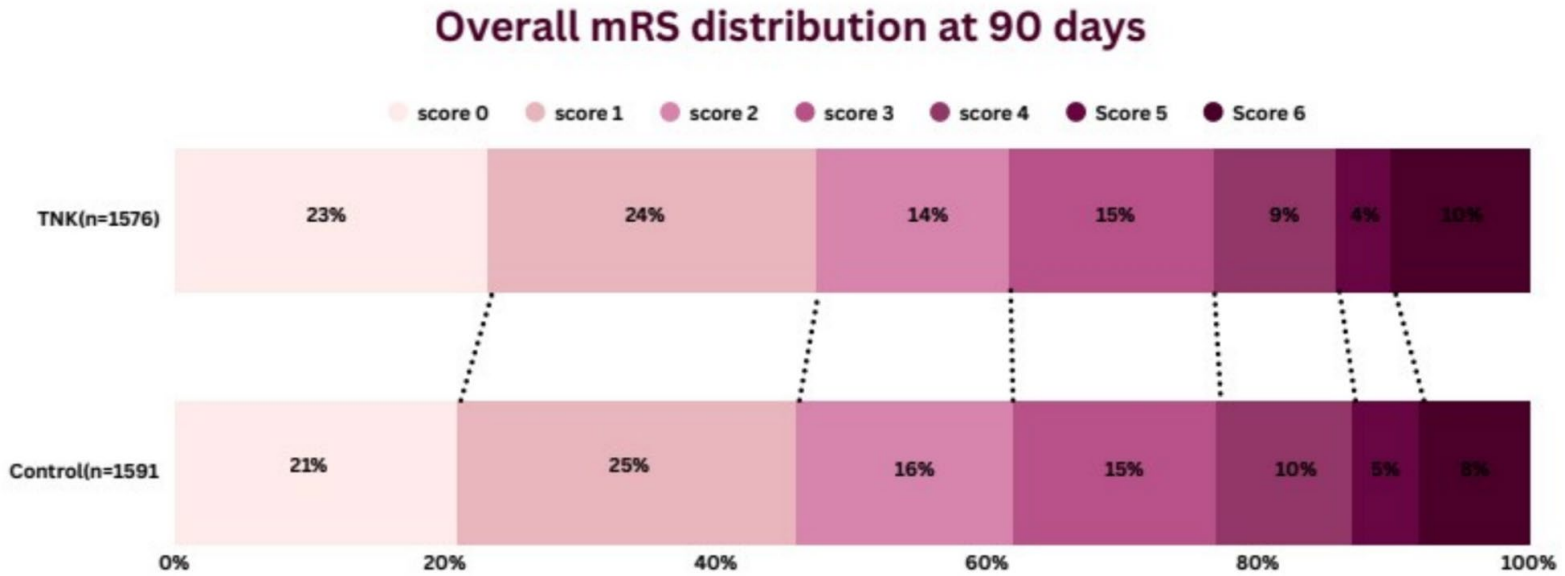
mRS distribution at 90 days

Excellent functional outcomes (mRs 0–1 at 90 days) were assessed in 8 studies, including 1533 patients in the intervention group and 1548 in the control group. No difference was observed between the two groups, as 735 patients achieved this outcome in the TNK 0.25 mg/kg compared to 701 in the control group (47.9% vs. 45.28%) (RR: 1.08, 95% CI [0.97–1.2], *P* value: 0.15, *I*^2^: 37%, Cochran *P* value: 0.13), see Table 2 and Fig. 2

**Table 2:** Meta-analysis results

Outcome	NO of studies	NO of patients in TNK group	NO of patients in control group	Risk ratio (RR)	95%CI	*P* value	Cochran Q *P* value (*I*^2^)
Main analysis							
Excellent functional outcomes (mRS 0–1 at 90 days)	8	735/1533 (47.95%)	701/1548 (45.2%)	1.08	[0.97, 1.20]	0.15	0.13 (37%)
Favorable functional outcomes (mRS 0–2 at 90 days)	8	944/1533 (61.58%)	951/1548 (61.43%)	1.02	[0.93, 1.11]	0.69	0.05 (50%)
Early neurological improvements	6	404/1002 (4.03%)	332/1024 (32.42%)	1.44	[1.06, 1.94]	0.02	0.005 (70%)
Death at 90 days	8	153/1533 (9.98%)	121/1548 (7.8%)	1.25	[0.95, 1.65]	0.12	0.26 (22%)
Symptomatic intracranial hemorrhage (sICH)	7	41/1493 (2.7%)	18/1508 (1.19%)	2.12	[1.21, 3.71]	0.008	0.76 (0%)
Poor functional outcomes (mRS 5–6 at 90 days)	8	213/1533 (13.8%)	195/1548 (12.6%)	1.09	[0.87, 1.35]	0.45	0.24 (23%)
Any intracranial hge (ICH)	3	65/449 (14.48%)	51/452 (11.28%)	1.25	[0.83, 1.9]	0.28	0.29 (20%)
Systemic bleeding	3	11/663 (1.66%)	9/655 (1.37%)	1.15	[0.48, 2.79]	0.75	0.41 (0%)
Parenchymal hematoma type 2 (PH-2)	5	33/779 (4.24%)	19/795 (2.39%)	1.73	[1, 3.02]	0.05	0.93 (0%)
Recanalization	3	184/603 (30.5%)	144/595 (24.2%)	1.65	[0.82, 3.30]	0.16	0.0001 (89%)
Reperfusion	3	265/603 (43.95%)	154/595 (25.9%)	2.35	[0.92, 6.05]	0.08	< 0.0001 (94%)
Subgroup Analysis according to mRS 0–2							
Excellent functional outcomes (mRS 0–1 at 90 days)	4	495/889 (55.68%)	475/916 (51.86%)	0.99	[0.9, 1.1]	0.91	0.43 (11%)
Favorable functional outcomes (mRS 0–2 at 90 days)	4	585/891 (65.7%)	633/917 (69%)	0.95	[0.9, 1]	0.04	0.51 (0%)
Early neurological improvements	3	343/662 (51.8%)	312/686 (45.48%)	1.15	[1.03, 1.28]	0.01	0.63 (0%)
Death at 90 days	4	86/891 (9.65%)	64/917 (6.98%)	1.45	[0.85, 2.48]	0.17	0.08 (56%)
Symptomatic intracranial hemorrhage (sICH)	4	36/891 (4%)	13/917 (1.42%)	1.89	[0.96, 3.73]	0.07	0.44 (0%)
Poor functional outcomes (mRS 5–6 at 90 days)	4	126/891 (14.14%)	110/917 (12%)	1.18	[0.85, 1.62]	0.32	0.18 (39%)
Subgroup Analysis according to mRS 0–1							
Excellent functional outcomes (mRS 0–1 at 90 days)	3	146/354 (41.24%)	115/341 (33.7$)	1.18	[1, 1.39]	0.06	0.38 (0%)
Favorable functional outcomes (mRS 0–2 at 90 days)	3	182/354 (51.4%)	145/341 (42.52%)	1.17	[1.02, 1.34]	0.03	0.37 (0%)
Early neurological improvements	3	61/354 (17.23%)	20/341 (5.87%)	2.99	[1.84, 4.85]	< 0.0001	0.7 (0%)
Death at 90 days	3	39/354 (11%)	34/341 (9.97%)	1.06	[0.68, 1.63]	0.81	0.43 (0%)
Symptomatic intracranial hemorrhage (sICH)	2	9/314 (2.9%)	2/301 (0.66%)	3.63	[0.91, 14.52]	0.07	0.88 (0%)
Poor functional outcomes (mRS 5–6 at 90 days)	3	53/354 (14.97%)	54/341 (15.83%)	1.18	[0.46, 2.98]	0.73	0.23 (32%)
Comparison between TNK 0.25 mg/kg versus best medical management							
Excellent functional outcomes (mRS 0–1 at 90 days)	6	624/1255 (51.16%)	606/1269 (47.76%)	1.07	[0.93, 1.22]	0.34	0.09 (48%)
Favorable functional outcomes (mRS 0–2 at 90 days)	6	799/1255 (63.67%)	817/1269 (64.38%)	1	[0.90, 1.11]	0.97	0.04 (57%)
Early neurological improvements	5	394/952 (41.39%)	330/975 (33.85%)	1.35	[1.02, 1.78]	0.04	0.01 (69%)
Death at 90 days	6	109/1255 (8.7%)	81/1269 (1.42%)	1.38	[0.92, 2.08]	0.12	0.12 (42%)
Symptomatic intracranial hemorrhage (sICH)	5	33/1215 (2.6%)	13/1229 (1.02%)	2.4	[1.25, 4.61]	0.009	0.6 (0%)
Poor functional outcomes (mRS 5–6 at 90 days)	6	151/1255 (12%)	136/1229 (10.7%)	1.13	[0.83, 1.56]	0.44	0.11 (45%)
Systemic bleeding	3	11/663 (1.66%)	9/655 (1.37%)	1.15	[0.48, 2.79]	0.75	0.41 (0%)
Parenchymal hematoma type 2 (PH-2)	4	25/551 (4.54%)	13/565 (2.3%)	1.92	[1, 3.68]	0.05	0.9 (0%)
Subgroup analysis (mechanical thrombectomy allowed)							
Excellent functional outcome	5	589/1179 (49.96%)	586/1207 (48.55%)	1.04	[0.93, 1.17]	0.5	0.17 (37%)
Poor functional outcome	5	160/1179 (13.57%)	141/1207 (11.68%)	1.14	[0.9, 1.45]	0.27	0.29 (19%)
Favorable functional outcome	5	762/1179 (64.63%)	806/1207 (66.78%)	0.96	[0.91, 1]	0.08	0.42 (0%)
Early neurological improvement	3	343/662 (51.8%)	312/686 (45.48%)	1.15	[1.03, 1.28]	0.01	0.63 (0%)
Symptomatic intracranial hge	5	32/1179 (2.7%)	16/1207 (1.33%)	1.91	[1.04, 3.52]	0.04	0.61 (0%)
Death	5	114/1179 (9.7%)	87/1207 (7.2%)	1.35	[0.93, 1.97]	0.12	0.15 (41%)
Subgroup analysis (Mechanical thrombectomy not allowed)							
Excellent functional outcome	3	146/354 (41.24%)	115/341 (33.7%)	1.18	[1.00, 1.39]	0.06	0.38 (0%)
Poor functional outcome	3	53/354 (14.97%)	54/341 (15.8%)	1.18	[0.46, 2.98]	0.73	0.23 (32%)
Favorable functional outcome	3	182/354 (51.4%)	145/341 (42.5%)	1.17	[1.02, 1.34]	0.03	0.37 (0%)
Early neurological improvement	3	61/340 (17.94%)	20/338 (8.88%)	2.99	[1.84, 4.85]	< 0.00001	0.70 (0%)
Symptomatic intracranial hge	2	9/314 (2.5%)	2/301 (0.66%)	3.63	[0.91, 14.52]	0.07	0.88 (0%)
Death	3	39/354 (11%)	34/341 (9.97%)	1.06	[0.68, 1.63]	0.81	0.43 (0%)

**Fig. 3:**
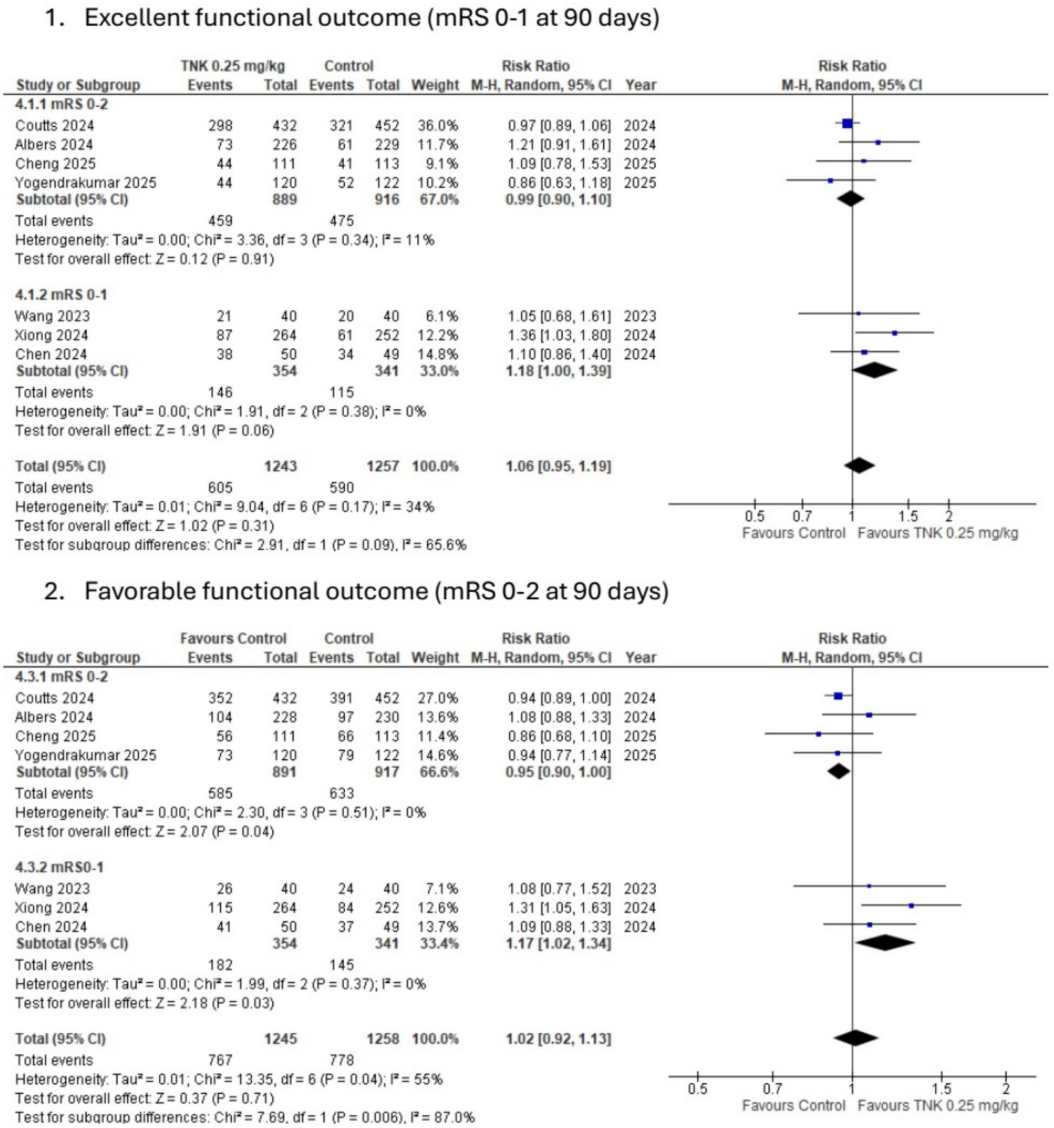
Subgroup analysis according to study baseline inclusion criteria of modified Rankin score (mRS) for 1/excellent functional outcomes 2/favorable functional outcomes

The same was in the favorable functional outcomes, as 8 studies assessed this outcome. 944 patients achieved this outcome in the TNK 0.25 mg/kg group versus 951 in the control group (61.6% vs. 61.4%). (RR: 1.02, 95% CI [0.93–1.11], *P* value: 0.69, *I*^2^: 50%, Cochran *P* value: 0.05). For early neurological improvement, there was a statistically significant increase in the TNK 0.25 mg/kg versus the control group (40.3% vs. 32.4%) (RR: 1.44, 95% CI [1.6–1.94], *P* value: 0.02, *I*^2^: 70%, Cochran *P* value: 0.005), see Table 2 and Fig. 3.

**Fig. 4:**
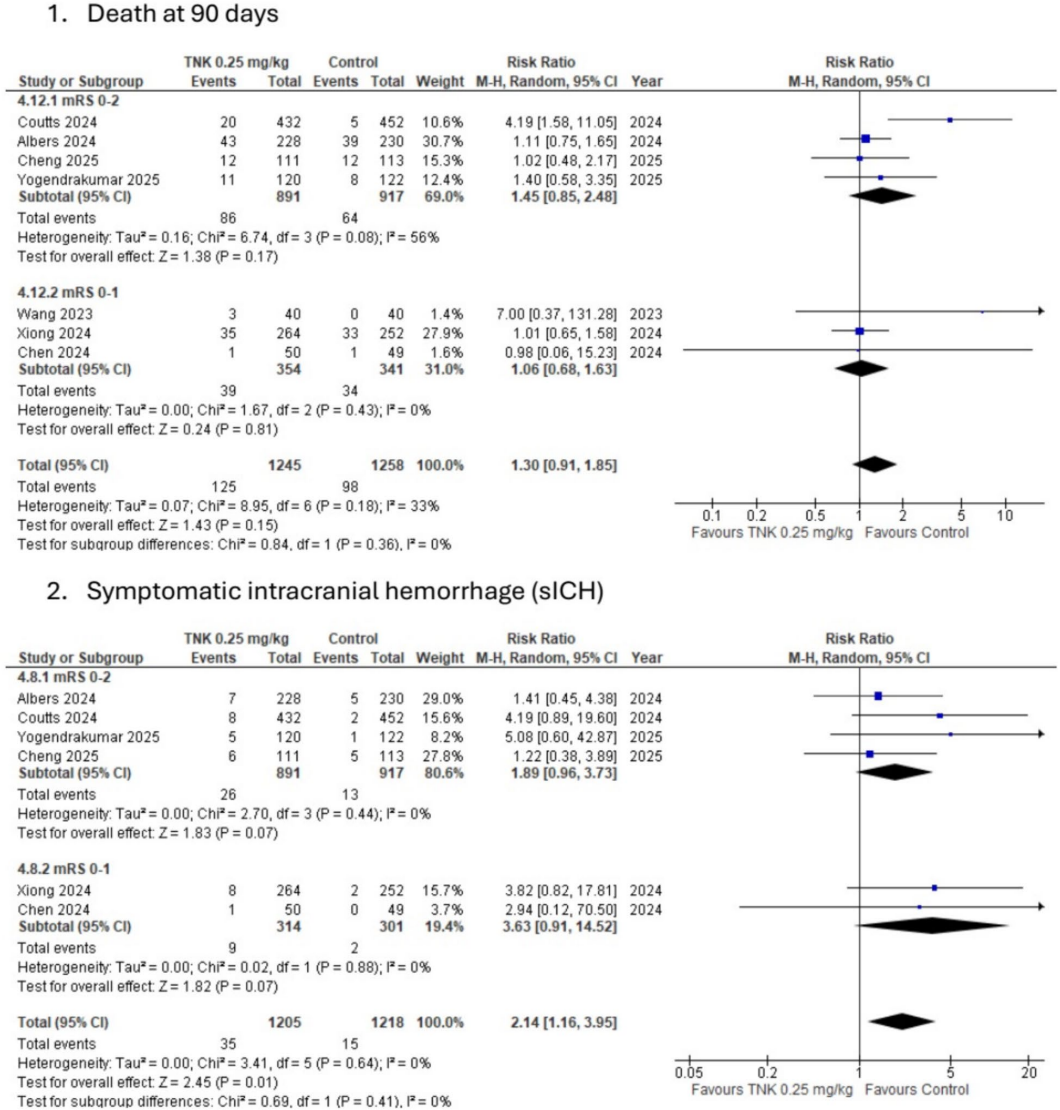
Subgroup analysis according to study baseline inclusion criteria of modified Rankin score (mRS) for 1/death 2/symptomatic intracranial hemorrhage (sICH)

#### Primary safety outcomes

The death rate was assessed in 8 studies. There was an increase in the death rate in the TNK 0.25 mg/kg group compared to the control group (10% vs. 7.8%); however, this increase did not reach statistical significance. (RR: 1.25, 95% CI [0.95, 1.65], *P* value: 0.12, *I*^2^: 22%, and Cochran *P* value: 0.26). On the other hand, there was a statistically significant increase in the sICH rate in the TNK 0.25 mg/kg compared to the control group (2.74% vs. 1.19%). (RR: 2.12, 95% CI [1.21–3.71], *P* value: 0.008, *I*^2^: 0%, and Cochran *P* value: 0.76), see Table 2 and (Fig. 4).

#### Secondary outcomes

For secondary outcomes, there was also no difference between the TNK 0.25 mg/kg and the control group in achieving reperfusion and recanalization rates (43.75% vs. 25.9%, RR 1.65, 95% CI [0.82–3.3], *P* value 0.16, *I*^2^: 89%, and Cochran *P* value: 0.0.0001), (30.5% vs. 24.2%, RR 2.35, 95% CI [0.92–6.05], *P* value 0.08, *I*^2^: 94%, and Cochran *P* value: < 0.0001) respectively, also, there was no statistically significant increase in parenchymal hematoma type 2 among TNK group (4.24% vs. 2.39%, RR 1.73, 95% CI [1.3–3.02], *P* value 0.05,, *I*^2^: 0%, and Cochran *P* value: 0.93), with an increase in both intracranial and systemic bleeding rates in the TNK group compared to the control group, see Table 2.

#### Subgroup analysis

When comparing patients based on baseline mRS, patients with mRS 0–1 who received TNK had a higher rate of favorable outcomes and early neurological improvement compared to the control group, while those with mRS 0–2 achieved lower rates of favorable outcomes and early neurological improvement compared to the control group. For favorable functional outcomes (51.4% vs. 42.52%, RR: 1.17, 95% CI [1.02, 1.34], *P* value: 0.03) and (65.7% vs. 69%, RR: 0.95, 95% CI [0.9, 1], *P* value: 0.04) for patients with mRs 0–1 and mRs 0–2, respectively. For early neurological improvement (17.23% vs. 5.87%, RR: 2.99, 95% CI [1.84, 4.85], *P* value: < 0.0001), and (51.8% vs. 45.48%, RR: 1.15, 95% CI [1.03, 1.28], *P* value: 0.01), see Table 2 and supplementary materials.

In addition, when assessing the effect of TNK compared to best medical management, excluding the studies that used a placebo as the comparator group. There was a significant increase in achieving early neurological improvement in TNK 0.25 mg/kg, and again, there was a statistically significant increase in symptomatic intracranial hemorrhage compared to the control group, see Table 2.

Finally, we performed a subgroup analysis on those patients who received MT compared to those who did not. When patients did not receive MT in the extended window, they had a higher chance of benefiting from TNK than in those studies in which they did receive MT. For example, patients who did not have MT had better excellent functional outcomes than the other group compared to the control group (41.24% vs. 33.7%, RR 1.18, 95% CI [1–1.39], *P* value 0.06) and (49.6% vs. 48.55%, RR 1.04, 95% CI [0.98–1.22], *P* value 0.62), respectively. In addition, for favorable functional outcomes, there was a statistically significant improvement for the studies that excluded these patients (RR 1.17, 95% CI [1.02–1.34], *P* value 0.03). However, the control group was better than the TNK group in the other subgroup (RR 0.96, 95% CI [0.9–1.03], *P* value 0.28), see Table 2. On the other hand, there was no difference in the subgroups in safety measures, see Table 2 and Supplementary material.

#### Sensitivity analysis

We performed a leave-one-out test to assess the effect of manual removal of each study on the heterogeneity and significance of the outcomes. Coutts 2024 was the source of heterogeneity for the favorable functional outcomes, as its removal changed the *I*^2^ from 50 to 24% and the Cochran Q *P* value from 0.05 to 0.25. In addition, its removal changed the *P* values of mRs 0–1 from 0.15 to 0.02. This may be because Coutts 2024 study included patients with a baseline NIHSS score of 0–5, while the rest included patients with NIHSS > 5. See supplementary material.

In addition, Albers 2024 removal changed the *P* values of both reperfusion and recanalization from 0.16 and 0.08 to 0.09 to 0.002, respectively. Finally, the removal of Xiong 2025 reduced the heterogeneity and *P* value of the early neurological improvement, as it changed from group (40.3% vs. 32.4%, RR: 1.44, 95% CI [1.6–1.94], *P* value: 0.02, *I*^2^: 70%, Cochran *P* value: 0.005), to (group (48.4% vs. 40.9%, RR: 1.32, 95% CI [0.87–1.56], *P* value: 0.09, *I*^2^: 52%, Cochran *P* value: 0.08).

#### Publication bias

As the included studies were only 9, we could not perform a publication bias analysis.

## Discussion

This study assesses the impact of using tenecteplase 0.25 mg/kg in patients with acute ischemic stroke in the extended time window from 4.5 to 24 h or more than 4.5 h since the last known well. Our findings revealed TNK 0.25 mg/kg improved only early neurological improvement with no significant difference in achieving excellent or functional outcomes compared to the control group. This appeared in the distribution of mRs at 90 days, as 47.9% of the TNK group patients had an mRS score of 0–1 compared to 45.28% in the control group, while 61.4% had an mRS score of 0–2 in the TNK group compared to 61.4%.

On the other hand, Safety measures were worse in the TNK group, as there was a statistically significant increase in symptomatic intracranial hemorrhage and parenchymal hematoma type 2 in the TNK group, respectively, and also an increase in death rates, with no statistically significant difference. These findings are consistent when analyzing only studies that compared TNK with the best medical management only.

When comparing studies with different inclusion criteria for baseline mRS, we found that studies including patients with mRS 0–1 had better outcomes than those with mRS 0–2 in all efficacy outcomes. Interestingly, there was a statistically significant increase in both favorable functional outcomes and early neurological improvements in patients with baseline mRS 0–1. On the other hand, safety measures were almost the same, but patients with baseline mRS 0–1 still had better outcomes than others. This may be because patients with mRs 0–1 before administering the drug had less disability than patients with mRs 0–2, so they are likely to have better outcomes after having TNK.

Finally, Studies that included only patients who would not undergo mechanical thrombectomy (MT) in addition to TNK had better outcomes than studies that included patients with assigned MT or who would perform it according to the disease progression. As there was a significant improvement in both favorable functional outcomes and early neurological improvement in the patients who did not undergo post-procedure MT. On the other hand, Patients who received MT in addition to TNK in the extended time window had a higher rate of symptomatic ICH, indicating that adding IV TNK 0.25 mg/kg before MT in the extended time window was associated with worse outcomes. This may be because adding IV TNK to MT would increase the hemorrhagic complication, because these patients would likely have larger occlusions and NIHSS scores. In addition, it may be because occlusion secondary to atherosclerosis makes the recanalization process take a longer time, which also plays a role in increasing hemorrhagic complications [45]. In addition, there was no difference in the death rates between the either in patients who had MT or not; however, patients with MT in addition to TNK experienced higher death rates compared to the control group (9.7% vs. 7.2%), while patients with TNK without MT experienced similar death rates compared to the control group (11% vs. 10%). This may be because of MT complications, such as procedure bleeding risk or arterial rupture, which increase the infarct growth, leading to an increase in death rates [45].

Previous systematic reviews had some similar findings to this study and some variations. Günkan et al. [46] is a recent systematic review that assessed the efficacy and safety of intravenous thrombolysis (IVT) in the extended time window for patients not willing to undergo mechanical thrombectomy. They included eight studies, of which 3 had tenecteplase [34, 38, 40], and the other 5 studies assessed the efficacy of alteplase. They found a statistically significant improvement in all efficacy measures, including excellent and favorable functional outcomes (mRS 0–1 and mRS 0–2, respectively) (53.56% vs. 36%, OR: 1.43 95% CI [1.17, 1.75], *P* value: 0.005), (57.4% vs. 50.5%, OR: 1.36 95% CI [1.12, 1.66], *P* value: 0.002) and early neurological improvement for IVT compared to the control group (18.8% vs. 10%, OR: 2.7, 95% CI [1.82, 4.01], *P* value: < 0.0001) while also an increase in both death and symptomatic intracranial hemorrhage rates in the IVT group (8.37% vs. 6.5%, OR: 1.28, 95% CI [0.87, 1.89], *P* value: 0.21) and (2.7% vs. 0.49%, OR: 4.2595% CI [1.67, 10.84], *P* value: 0.002). They differ from our results in efficacy outcomes, because they include both alteplase and tenecteplase, and also, they include studies that did not include patients willing to undergo mechanical thrombectomy. However, their results are the same as ours in safety measures.

They performed a subgroup analysis to assess the effect of TNK only. They found a statistically significant improvement in the mRs 0–1 and mRs 0–2 with an increase in both death and sICH without a statistically significant increase. This variation in the outcomes is because they only included three studies and used odds ratios and weighted their results using inverse variance, while we used Risk ratios and weighted our results using the default RevMan setting (Mantel–Haenszel).

Al-Janabi et al. [47] was another meta-analysis that compared IV thrombolysis using TNK and alteplase versus medical management in patients with AIS in the extended time window. Their results were similar to Günkan et al. 2025. As Al-Janabi et al. 2024 also found a statistically significant increase in both mRs 0–1 and 0–2, while there was no difference in mortality rates; however, they did not report sICH results.

On the other hand, Palaiodimou et al. [23] was an earlier systematic review that included only 3 randomized controlled trials about the efficacy of tenecteplase in the extended time window. They found a statistically significant improvement in the excellent functional outcomes (40.4% vs. 34.35%, RR: 1.17, 95% CI [1.01, 1.36], *P* value:0.04) for tenecteplase over the control group and an increase in death and symptomatic intracranial hemorrhage in the TNK group, with no statistical significance (13.54% vs. 12%, RR: 1.1, 95% CI [0.81, 1.49], *P* value:0.53) and (2.38% vs. 1.432%, RR: 1.67, 95% CI [0.7, 4], *P* value: 0.25), although their results cannot be inclusive due to the small studies included. Aladawi et al. [48] was another systematic review in the same scope. They also included 3 studies, as Palaiodimou et al. 2024 and had the same findings as Palaiodimou et al. 2024.

Cheng [37] was an RCT that compared two doses of TNK (0.25 mg/kg vs. 0.32 mg/kg). They found that TNK 0.25 mg/kg was better in achieving early perfusion without sICH. They also found similar results in sICH, as in both groups, only 4 had sICH. On the other hand, TNK 0.25 mg/kg was worse in infract growth at 3–5 days compared to TNK 0.32 mg/kg, and also TNK 0.25 mg/kg was associated with an increase in parenchymal hematoma.

Unlike other included RCTs, Coutts [36] included patients with baseline NIHSS scores of more than 5. Their results were different from other studies, as they found that TNK was worse in achieving mRs 0–1 and mRs 0–2, as about 69% achieved mRs 0–1 in the TNK group compared to 71% in the control group, and 81% in the TNK group achieved mRs 0–2 compared to 87% in the control group. RR 0·97 (0·90–1·06) and RR 0·94 (0·89–1·00), respectively. For the safety measures, there was an increase in death and symptomatic intracranial hemorrhage in the TNK group compared to the control group. (5% vs. 1%, HR 3·8 (1·4 to 10·2), and (2% vs. < 1%, *P* value: 0.059). This variation in the baseline of Coutts 2024 compared to other studies was observed in the sensitivity analysis, as its removal changed the *P* value of mRs 0–1 from 0.15 to 0.02, and reduced the heterogeneity of mRs 0–2 (*I*^2^ changed from 50 to 24%, and Cochran’s *P* value changed from 0.05 to 0.25).

Several trials are now testing TNK’s efficacy and safety as an intra-arterial thrombolytic after mechanical thrombectomy. Hu [49] and Hung [50] are recent trials published on this topic, and their results revealed that patients who receive TNK have a higher likelihood of achieving an excellent outcome and early neurological improvement, but did not reach statistical significance. However, they also found an increase in symptomatic intracranial hemorrhage rates in the TNK group and a statistically significant increase in any intracranial hemorrhage. In addition, in a recent systematic review that included 7 RCTs, Jiang et al. [51] showed that adjuvant intra-arterial TNK to mechanical thrombectomy was superior to mechanical thrombectomy alone in achieving mRs 0–1 with no significant difference in death or sICH rates.

Despite these positive results of TNK, the small number of studies limits its generalizability, so it is important to conduct several trials, especially to assess the subgroup analysis results. Fortunately, there are several trials conducted now to assess these effects, such as DIRECT–TNK (NCT05199194), POST-ETERNAL (NCT05105633), TRACE Ⅳ (NCT06414499), and EXTEND-IV (NCT05199662).

### Strengths and limitations

This study has several limitations. First, a small number of the included articles limits the generalizability of the results. In addition, subgroup analysis based on the study inclusion criteria of mRS limits the number of studies in each subgroup, making it difficult to determine mRS’s effect on the TNK results. Again, we cannot be sure if the inclusion of patients who would have had mechanical thrombectomy in addition to TNK had a negative impact on the study results, or if it was just an association. Most studies have included patients based on radiological findings, not all patients with an acute ischemic stroke, which again limits their generalizability. Finally, Interstudy heterogeneity limits the primary study findings.

On the other hand, this study has several strengths, as it is the most updated study assessing the efficacy and safety of TNK 0.25 mg/kg in acute ischemic stroke in the extended time window. Most of the subgroup analyses are homogeneous, making it applicable to rely on their results. In addition, we shed light on the side effects of TNK, especially symptomatic intracranial hemorrhage.

## Conclusion

There is evidence that there is no benefit of TNK in the extended time window, but certain subpopulations, such as those with mRS 0–1, do benefit. For safety measures, TNK 0.25 appears to significantly increase the risk of symptomatic intracranial hemorrhage. However, no difference in safety measures was observed in patients with extended time window ischemic stroke whose baseline mRs ranged from 0 to 1. In addition, there was a significant improvement in favorable functional outcomes in patients not willing to perform MT. These findings support using TNK0.25 mg/kg for patients with extended time window ischemic stroke who will not undergo mechanical thrombectomy in addition to TNK or whose baseline mRs ranges from 0 to 1. Several randomized controlled trials should be conducted to further assess its use, especially in patients with minor ischemic stroke, and test its side effects. Finally, TNK may be an effective alternative in patients with an extended time of stroke and not fit for mechanical thrombectomy, or MT is not available for them.

## Supplementary Information

Additional file 1.

## Data Availability

Data is available in the electronic version of this article and the attached supplementary material.
