# Supplementary material for: Safety and efficacy of intravenous tenecteplase in patients with acute ischemic stroke in extended time window: systematic review and meta-analysis
Source: Eur J Med Res. 2025 Nov 29;30:1203. doi: 10.1186/s40001-025-03466-7 (PMC12670736; doi:10.1186/s40001-025-03466-7)
Supplement: Supplementary file 1 — Additional file 1. [file 40001_2025_3466_MOESM1_ESM.pdf]

Supplementary materials

Systematic review and meta-analysis

Contents

Search Strategy....1
S1: inclusion criteria and main findings....2
Table S2: baseline catachrestic Part 1....0
Table S2: baseline catachrestic Part 2....2
Table S3: Sensitivity analysis results....0
Meta-analysis results....1
Main analysis....1
Subgroup analysis according to the inclusion criteria of mRS 3
comparison between TNK 0.25 mg/kg vs best medical management....7
Subgroup analysis according to the allowance of post-procedure mechanical thrombectomy (MT)....9
mRS distribution at 90 days....12
Quality assessment results....17

Search Strategy

Database	Search strategy
PubMed	(Ischemic stroke OR Ischaemic Stroke OR Acute Ischemic Stroke OR Cryptogenic Ischemic Stroke OR Cryptogenic Embolism Stroke OR AIS OR transient ischemic attack OR TIA) AND (Tenecteplase OR Metalyse OR TNKase OR TNK OR Elaxim).
Scopus	(“Ischemic stroke” OR “Ischaemic Stroke” OR “Acute Ischemic Stroke” OR “Cryptogenic Ischemic Stroke” OR “Cryptogenic Embolism Stroke” OR “transient ischemic attack”) AND (Tenecteplase OR Metalyse OR TNKase OR TNK OR Elaxim).
Web of Science	(Ischemic stroke OR Ischaemic Stroke OR Acute Ischemic Stroke OR Cryptogenic Ischemic Stroke OR Cryptogenic Embolism Stroke OR AIS OR transient ischemic attack OR TIA) AND (Tenecteplase OR Metalyse OR TNKase OR TNK OR Elaxim).

S1: inclusion criteria and main findings		
Study ID	Inclusion criteria	Main findings
Roaldsen 2023	1. aged 18 years or older<br>2. acute ischemic wake up stroke<br>3. stroke symptoms upon awakening that were not present before sleep<br>4. Limb weakness<br>5. National Institutes of Health Stroke Scale (NIHSS) score of 3 or higher or aphasia<br>6. Non-contrast CT examination of the head<br>7. The ability to receive tenecteplase within 4·5 h of awakening.	• Treatment with tenecteplase was not associated with better functional outcomes at 90 days.<br>• Patients with an excellent functional outcome were more in the tenecteplase group than the control group.<br>• Patients with good functional outcomes were the same between treatment groups.<br>• Median scores on the MMSE, Barthel Index, median change in NIHSS from baseline to 24 h and 7 days, and the EQ-5D-VAS at 90 days were similar between treatment groups.<br>• Mortality at 90 days did not significantly differ between treatment groups within 90 days Symptomatic intracranial hemorrhage was higher in the tenecteplase group
Wang 2023	1. aged 18 to 80 years<br>2. acute moderate to severe ischemic stroke<br>3. National Institutes of Health Stroke Scale [NIHSS] scores 6 to 25 at admission<br>4. Functioning independently in the community (modified Rankin Scale [mRS] scores 0 to 1)<br>5. The time window is 4.5–24 hours after the onset of stroke symptoms<br>6. Non-contrast computed tomography screening to exclude hemorrhagic stroke.	• There was no significant difference between the two groups in excellent functional outcome at 90 days (modified Rankin Scale [mRS] score of 0–1).<br>• More early neurological improvement occurred in the TNK group than in the control group.<br>• There were no significant differences in secondary endpoints: such as mRS 0–2 at 90 days, shift analysis of mRS at 90 days, and change in National Institutes of Health Stroke Scale score at 24 hours and 7 days.<br>• There were no cases of sICH in this trial; however, asymptomatic intracranial hemorrhage was higher in the tenecteplase group.
Albers 2024	1. at least 18 years of age<br>2. independent function before the stroke (baseline pre-stroke modified Rankin scale score, 0 to 2)<br>3. Had an ischemic stroke<br>4. Could receive tenecteplase or placebo 4.5 to 24 hours after the time they were last known to be well<br>5. National Institutes of Health Stroke Scale (NIHSS) score of at least 5 that was attributed to occlusion of the internal carotid artery (either cervical or intracranial) or the M1 or M2 segment of the middle cerebral artery or both, on CT angiography or magnetic resonance angiography.<br>6. Evidence of salvageable brain tissue from CT perfusion imaging or perfusion diffusion MRI.	• There was no significant improvement in functional outcomes at 90 days in patients who received Tenecteplase.<br>• The incidence of recanalization at 24 hours appeared to be higher with tenecteplase than with placebo, but the incidence of reperfusion was similar in the two groups at the end of the procedure.<br>• Mortality at 90 days did not differ appreciably between the two trial groups; the incidence of symptomatic intra-cerebral hemorrhage was similar in the two groups.<br>• The incidence of adverse events, serious adverse events, and withdrawal from the trial due to

	7. Tenecteplase or placebo was given as soon as possible, ideally before the arterial puncture for a planned endovascular thrombectomy.	adverse events did not differ appreciably between the groups.<br>• Complete recanalization as assessed on 24-hour angiography by MRI or CT appeared to be higher in the tenecteplase group while as reperfusion was similar in the two groups
Cheng 2024	1. Acute ischemic stroke within 4.5–24 hours from time of last seen well and were aged 18 years or older<br>2. Clinically significant acute neurological deficit measured by the baseline National Institutes of Health Stroke Scale (NIHSS) score<br>3. Pre-stroke modified Rankin Scale (mRS) 0–2<br>4. Fulfilled the "dual target" imaging criteria, on baseline multimodal CT imaging and a favorable penumbral profile on CTP imaging.	• Patients in the 0.32 mg/kg tenecteplase stratum reached major reperfusion without sICH after tenecteplase thrombolysis more than 0.25 group.<br>• Recanalization was the same in the two groups.<br>• Excellent functional outcome (mRS 0–1) was achieved more in the 0.32 group, sICH was equal in the two groups
Coutts 2024	1. 18 years or older<br>2. Functionally independent before the stroke (baseline pre-stroke mRS 0–2)<br>3. Minor stroke with NIHSS score of 0–5<br>4. Presented within 12 h of last seen normal<br>5. Direct imaging evidence of an intracranial occlusion or indirect evidence of occlusion with a focal perfusion lesion relevant to the presenting symptoms<br>6. No region of well-evolved infarction concordant with the acute presenting syndrome and an Alberta Stroke Program Early CT score (ASPECTS)21 of 7 or greater<br>7. Perfusion imaging was not mandatory.	• More patients had an NIHSS of 0 in the tenecteplase group versus control<br>• Recanalization rates overall were higher in the tenecteplase-treated patients than in the control group, but this did not translate into improved functional outcomes at 90 days.<br>• More symptomatic intracranial hemorrhages occurred in the tenecteplase group.<br>• No extracranial hemorrhages were temporally related to treatment.<br>• There were more deaths in the tenecteplase group than in the control group
Chen 2024	1. adults aged 18 to 80 years<br>2. acute moderate to severe ischemic stroke [NIHSS] scores ≥ 4 at admission<br>3. functioning independently in the community (modified Rankin Scale [mRS] scores 0 to 1<br>4. enrolled within 4.5–6 h selected using NCCT or MRI	• intravenous TNK following NBP seemed safe, feasible, and might improve early neurological outcomes in acute ischemic stroke patients presenting within 4.5–6 h of onset selected using NCCT

Xiong 2024	1. 18 years of age or older<br>2. Patients with Stroke, including stroke on awakening and un-witnessed stroke, were recruited within 4.5 to 24 hours after the time that they were last known to be well.<br>3. Pre-stroke score of 0 or 1 on the modified Rankin scale<br>4. National Institutes of Health Stroke Scale [NIHSS] scores 6 to 25 at admission<br>5. Evidence of occlusion of the intracranial internal carotid artery or the first (M1) or second (M2) segment of the middle cerebral artery on computed tomographic (CT) angiography or magnetic resonance angiography<br>6. Evidence of salvageable brain tissue as identified on perfusion imaging	• Treatment with tenecteplase resulted in less disability of patients with a modified Rankin scale score of 0 or 1 at 90 days than standard medical treatment.<br>• The percentage of patients who were functionally independent (defined as a modified Rankin scale score of ≤2) at 90 days was higher in tenecteplase group than control group.<br>• Major neurological improvement and reperfusion at 24 h were higher in tenecteplase patients than control<br>• NIHSS score improvement was more in tenecteplase group than control group there was no marked difference in survival at 90 days.<br>• The incidence of symptomatic intracranial hemorrhage within the first 36 hours after treatment appeared to be higher in the tenecteplase group than in the standard treatment group.
Cheng 2025	1. Aged 18 to 80<br>2. Premorbid modified Rankin Scale (mRS) of 0 to 2.<br>3. They presented with AIS within 4.5 to 24 hours after the last known well<br>4. Clinically significant acute neurological deficit, as judged by the investigators and quantified by a National Institutes of Health Stroke Scale (NIHSS) score.<br>5. Meet the dual-target criteria on baseline multimodal imaging. The dual-target criteria were defined as large or medium vessel occlusion in the anterior circulation on baseline computed tomography angiography (CTA), and a favorable penumbral mismatch profile on baseline computed tomography perfusion (CTP).<br>6. Large or medium vessel occlusion encompassed occlusions in the extracranial or intracranial segments of the internal carotid artery, the first or second segments of the middle cerebral artery, and the first or second segments of the anterior cerebral artery on CTA.	• Treatment with tenecteplase resulted in a higher incidence of reperfusion without sICH, compared with the best medical treatment without tenecteplase thrombolysis.<br>• No significant difference was shown between treatment groups in secondary outcomes, including NIHSS at 24 to 48 hours, infarct growth at 3 to 5 days, or mRS score at 90 days. Therefore, it seems that the improved reperfusion did not transform into radiological and clinical benefits in the tenecteplase group, one of the major possible reasons for the neutral secondary outcome results may be the characteristics of our patient population
Yogendrakumar 2025	1. ≥18 years<br>2. anterior circulation LVO ischemic stroke presented within 24 hours of stroke onset<br>3. the presence of salvageable tissue without a large ischemic core	• The study did not show superiority of tenecteplase in patients with LVO in the entire reperfusion treatment time window (0–24 hours) when compared with standard of care

Table S2: baseline catachrestic Part 1

Study ID	Study Arms	Age	Sex (male)	Race						Occlusion sites						
				White	Black	Asian	Pacific islander	Others	Baseline NIHSS score	Internal segment of internal carotid artery	External segment of internal carotid artery	M1 segment of MCA	M2 segment of MCA	M3 segment of MCA	Anterior cerebral artery	Others
Roaldsen 2023	TNK	72.7±11.3	164 (57%)				6 (2%)	282 (98%)	6 [5-11]	69/231 (30%)						
	Best medical management	72.9±11.6	168 (58%)				8 (3%)	282 (97%)	6 [5-10]	83/226 (37%)						
Wang 2023	TNK	62.68±8.87	31 (77.50%)			40 (50%)			7.5 [6-10.75]	24 (60%)						16 (40%)
	Best medical management	62.8±8.56	26 (65%)			40 (50%)			7 [6.00-8.75]	28 (70%)						12 (30%)
Albers 2024	TNK	71±12.68	106 (46.50%)	169 (74.1 %)	31 (13.6%)	11 (4.8%)	2 (0.9%)	15 (6.6%)	12 [8–17]	20 (8.8%)		110 (48.2%)	89 (39%)			9 (3.9%)
	Placebo	72.67±14.17	107 (46.50%)	170 (73.9 %)	32 (13.9%)	9 (3.9%)	3 (1.3%)	14 (6.1%)	12 [8–18]	17 (7.4%)		117 (50.9%)	84 (36%)			12 (5.2%)
Cheng 2024	TNK	68.3±13.1	25 (58.10%)			43 (50%)			11 [8-15]	3 (7%)	3 (7%)	25 (58.1%)	9 (20.9%)		3 (7%)	0 (0%)
	(0.25mg/kg)	67.1±11.5	31 (72.10%)			43 (50%)			9 [6-13]	4 (9.3%)	6 (14 %)	15 (34.9%)	11 (25.6%)		6 (14%)	1 (2.3%)
Coutts 2024	TNK	71.33±13.4	244 (56%)	371 (86%)	6 (1%)	40 (9%)	1 (<1%)	10 (2%)	2 [1–3]							
	(0.32mg/kg)	70.67±13.4	272 (60%)	382 (85%)	7 (2%)	42 (9%)		16 (4%)	2 [1–3]							
Chen 2024	TNK 0.25 mg/kg and NBP	63.7±9.6	37(74%)						5[4-7							
	NBP alone	64.8±10.25	28(57.1%)						5[4-6]							
Xiong 2024	TNK	66.67±12.67	183 (69.30%)			264 (51.16%)			11 [7-15]	87(33%)		119 (45.1%)	58 (22%)			
	Best medical management	67.67±12.68	167 (66.30%)			252 (48.84%)			10 [7-14]	84(33.33%)		130 (51.6%)	38 (15.1%)			
Cheng 2025	TNK	64.2±10.4	80 (72.10%)			111 (49.55%)			9 [5-14]	9 (9.9%)	16 (14.4%)	53 (47.8%)	21 (18.9%)		10 (9%)	9 (9.9%)
	Best medical management	63.6±11	80 (70.80%)			113 (50.45%)			9 [6-16]	7 (6.2%)	21 (18.60%)	55 (48.7%)	24 (21.2%)		6 (5.3%)	7 (6.2%)

Yogendrakumar 2025	TNK	72.67±12.67	66 (55%)							19 (16%)	62(75.6%)	33 (28%)			5 (4%)
	Medical management	71±12.75	72 (59%)							20 (16%)	71(58%)	26 (21%)			5 (4%)

Table S2: baseline catachrestic Part 2

Study ID	Study Arms	Risk factors					Time from to (hour)		
		Hypertension	Diabetes	Atrial fibrillation/ Arrhythmia	Smoking	Previous stroke or TIA	last known well to randomization	Randomization to administration of the drug	Hospital arrival to administration of drug
Roaldsen 2023	TNK	176/276 (64%)	55/278 (20%)	55/267 (21%)	51/240 (21%)	75/277 (27%)	10.87 [9.22-12.9]		0.93 [0.72-1.33]
	Best medical management	177/279 (63%)	52/281 (19%)	31/272 (11%)	46/229 (20%)	60/274 (22%)	10.88 [8.73-12.58]		
Wang 2023	TNK	24 (60%)	9 (22.5%)		19 (47.5%)	11 (27.5%)			1.29 [0.97-1.88]
	Best medical management	28 (70%)	13 (32.5%)		21 (52.5%)	12 (30%)			1.3 [1.03-2.41]
Albers 2024	TNK						12.3 [9.2–15.6]	0.22 [0.12-0.33]	
	Placebo						12.7 [8.7–16.5]	0.23 [0.12-0.33]	
Cheng 2024	TNK	27 (62.8%)	12 (27.9%)	14 (32.6%)	15 (34.9%)	6 (14%)			2.17 [1.65-2.65]
	(0.25mg/kg)	29 (67.4%)	16 (37.2%)	4 (9.3%)	25 (58.1%)	5 (11.6%)			2.33 [1.85-3.62]
Coutts 2024	TNK	265 (61%)	82 (19%)	91 (21%)	172 (40%)	72 (17%)	4.77 [2.68–7.33]		
	(0.32mg/kg)	261 (58%)	86 (19%)	78 (17%)	176 (39%)	85 (19%)	4.55 [2.70–7.47]		
Chen 2024	TNK 0.25 mg/kg and NBP	32(64%)	15(30%)		24(48%)	13(32%)			
	NBP alone	29(59.2%)	15(30.6%)		18(36.7%)	18(36.7%)			
Xiong 2024	TNK	177 (67%)	69 (26.1%)	49 (18.6%)			12.3 [8.5 –16.4]		
	Best medical management	180 (71.4%)	71 (28.2%)	48 (19%)					
Cheng 2025	TNK	71 (64%)	32 (28.8%)	9 (8.1%)		15 (13.5%)			1.9 [1.32-2.67]
	Best medical management	79 (69.9%)	26 (23%)	20 (17.7%)		19 (16.8%)			1.6 [1.31-1.88]
	TNK	80(66.67%)		22 (18%)		0	193 (119–439)		

Yogendrakumar 2025	Medical management	67(54.9%)				1(1%)	177 (121–578)		

Sensitivity analysis results

Table S3: Sensitivity analysis results

Outcome	P value	Risk ratio	95%CI	P-value for Cochran's Q (I^2)
Excellent functional outcomes (mRS 0-1 at 90 days)	0.15	1.08	[0.97-1.2]	0.13 (37%)
Sensitivity analysis after removal of Coutts 2024	0.02	1.13	[1.02, 1.26]	0.51(0%)
Favorable functional outcomes (mRS 0-2 at 90 days)	0.69	1.02	[0.93-1.11]	0.05 (50%)
Sensitivity analysis after removal of Coutts 2024	0.34	1.04	[0.96, 1.14]	0.25 (24%)
Major neurological improvements	0.02	1.44	[1.06-1.94]	0.005 (70%)
Sensitivity analysis after removal Xiong 2024	0.09	1.32	[0.87, 1.56]	0.08 (52%)
Reperfusion	0.16	1.65	[0.82-3.3]	0.0001 (89%)
Sensitivity analysis after removal of Albers 2024	0.009	2.23	[1.22, 4.06]	0.1 (65%)
Recanalization	0.08	2.35	[0.92, 6.05]	<0.0001(94%)
Sensitivity analysis after removal of Albers 2024	0.0002	3.4	[1.8, 6.41]	0.09 (65%)

Meta-analysis results

Main analysis

1. Excellent functional outcomes (mRS 0-1 at 90 days)

Study or Subgroup	TNK 0.25 mg/kg Events	TNK 0.25 mg/kg Total	Control Events	Control Total	Weight	Risk Ratio M-H, Random, 95% CI	Year
Roaldsen 2023	130	288	111	290	16.3%	1.18 [0.97, 1.43]	2023
Wang 2023	21	40	20	40	5.1%	1.05 [0.68, 1.61]	2023
Coutts 2024	298	432	321	452	29.7%	0.97 [0.89, 1.06]	2024
Albers 2024	73	228	61	230	9.8%	1.21 [0.91, 1.61]	2024
Xiong 2024	87	264	61	252	10.2%	1.36 [1.03, 1.80]	2024
Chen 2024	38	50	34	49	12.4%	1.10 [0.86, 1.40]	2024
Yogendrakumar 2025	44	120	52	122	8.6%	0.86 [0.63, 1.18]	2025
Cheng 2025	44	111	41	113	7.7%	1.09 [0.78, 1.53]	2025
**Total (95% CI)**		**1533**		**1548**	**100.0%**	**1.08 [0.97, 1.20]**	
Total events	735		701				

Heterogeneity: Tau² = 0.01; Chi² = 11.14, df = 7 (P = 0.13); I² = 37%
Test for overall effect: Z = 1.43 (P = 0.15)

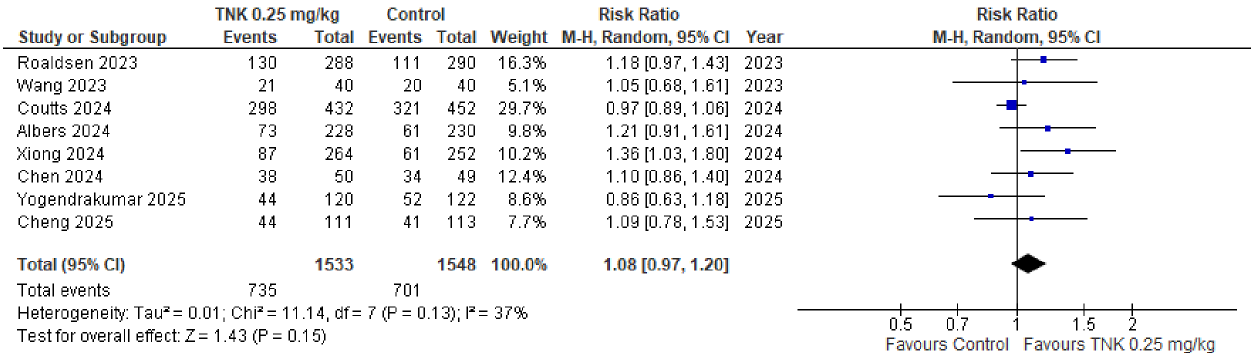

2. Favorable functional outcomes (mRS 0-2 at 90 days)

Study or Subgroup	TNK 0.25 mg/kg Events	TNK 0.25 mg/kg Total	Control Events	Control Total	Weight	Risk Ratio M-H, Random, 95% CI	Year
Wang 2023	26	40	24	40	5.2%	1.08 [0.77, 1.52]	2023
Roaldsen 2023	177	288	173	290	17.2%	1.03 [0.90, 1.18]	2023
Chen 2024	41	50	37	49	10.9%	1.09 [0.88, 1.33]	2024
Coutts 2024	352	432	391	452	25.6%	0.94 [0.89, 1.00]	2024
Xiong 2024	115	264	84	252	9.8%	1.31 [1.05, 1.63]	2024
Albers 2024	104	228	97	230	10.8%	1.08 [0.88, 1.33]	2024
Yogendrakumar 2025	73	120	79	122	11.6%	0.94 [0.77, 1.14]	2025
Cheng 2025	56	111	66	113	8.8%	0.86 [0.68, 1.10]	2025
**Total (95% CI)**		**1533**		**1548**	**100.0%**	**1.02 [0.93, 1.11]**	
Total events	944		951				

Heterogeneity: Tau² = 0.01; Chi² = 13.98, df = 7 (P = 0.05); I² = 50%
Test for overall effect: Z = 0.40 (P = 0.69)

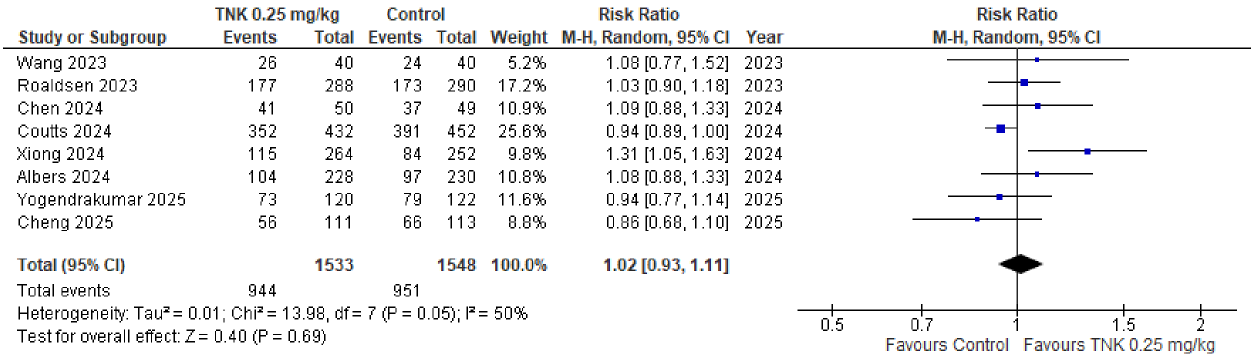

3. Major neurological improvements

Study or Subgroup	TNK 0.25 mg/kg Events	TNK 0.25 mg/kg Total	Control Events	Control Total	Weight	Risk Ratio M-H, Random, 95% CI	Year
Wang 2023	11	40	3	40	5.3%	3.67 [1.11, 12.16]	2023
Xiong 2024	40	250	15	249	15.1%	2.66 [1.51, 4.68]	2024
Coutts 2024	247	432	226	452	30.9%	1.14 [1.01, 1.29]	2024
Chen 2024	10	50	2	49	3.7%	4.90 [1.13, 21.23]	2024
Cheng 2025	24	110	26	112	17.6%	0.94 [0.58, 1.53]	2025
Yogendrakumar 2025	72	120	60	122	27.3%	1.22 [0.97, 1.54]	2025
**Total (95% CI)**		**1002**		**1024**	**100.0%**	**1.44 [1.06, 1.94]**	
Total events	404		332				

Heterogeneity: Tau² = 0.07; Chi² = 16.77, df = 5 (P = 0.005); I² = 70%
Test for overall effect: Z = 2.35 (P = 0.02)

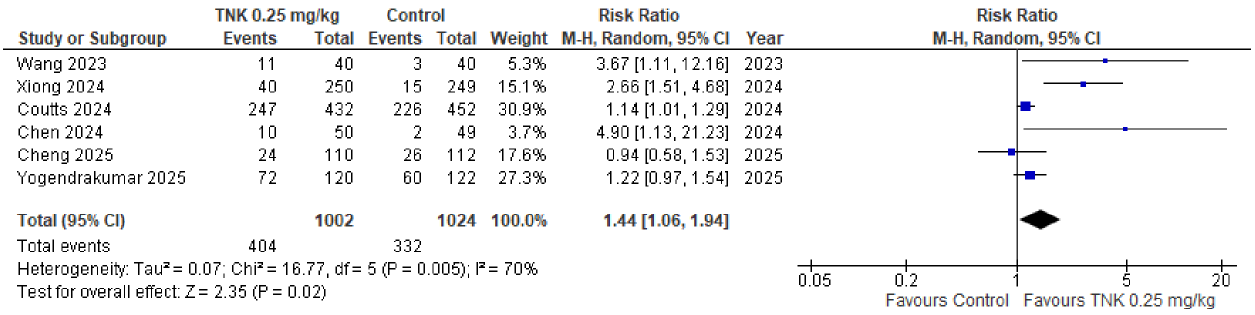

4. Reperfusion

Study or Subgroup	TNK 0.25 mg/kg Events	TNK 0.25 mg/kg Total	Control Events	Control Total	Weight	Risk Ratio M-H, Random, 95% CI	Year
Xiong 2024	48	264	27	252	33.2%	1.70 [1.09, 2.63]	2024
Albers 2024	99	228	105	230	37.0%	0.95 [0.78, 1.17]	2024
Cheng 2025	37	111	12	113	29.8%	3.14 [1.73, 5.70]	2025
**Total (95% CI)**		**603**		**595**	**100.0%**	**1.65 [0.82, 3.30]**	
Total events	184		144				

Heterogeneity: Tau² = 0.33; Chi² = 18.29, df = 2 (P = 0.0001); I² = 89%
Test for overall effect: Z = 1.41 (P = 0.16)

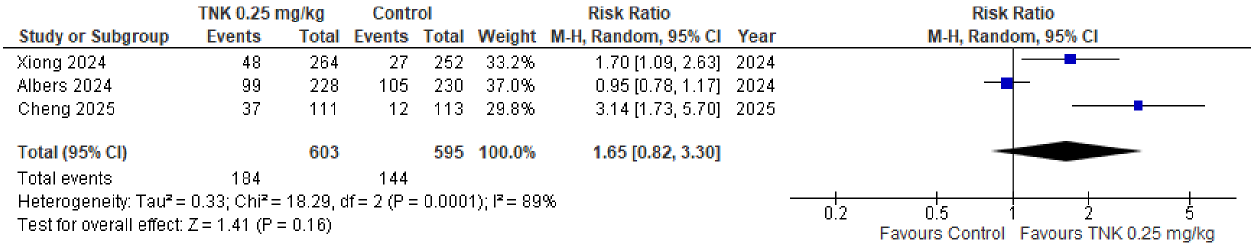

5. Recanalization

Study or Subgroup	TNK 0.25 mg/kg Events	TNK 0.25 mg/kg Total	Control Events	Control Total	Weight	Risk Ratio M-H, Random, 95% CI
Albers 2024	148	228	124	230	35.6%	1.20 [1.03, 1.40]
Cheng 2025	39	111	16	113	32.4%	2.48 [1.48, 4.17]
Xiong 2024	69	264	14	252	32.0%	4.70 [2.72, 8.14]
**Total (95% CI)**		**603**		**595**	**100.0%**	**2.35 [0.92, 6.05]**
Total events	256		154			

Heterogeneity: Tau² = 0.65; Chi² = 33.22, df = 2 (P < 0.00001); I² = 94%
Test for overall effect: Z = 1.78 (P = 0.08)

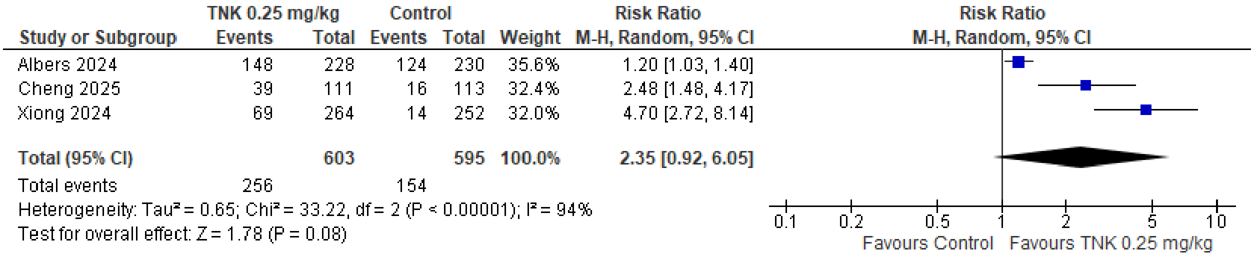

6. Death at 90 days

Study or Subgroup	TNK 0.25 mg/kg Events	TNK 0.25 mg/kg Total	Control Events	Control Total	Weight	Risk Ratio M-H, Random, 95% CI	Year
Wang 2023	3	40	0	40	0.9%	7.00 [0.37, 131.28]	2023
Roaldsen 2023	28	288	23	290	19.2%	1.23 [0.72, 2.08]	2023
Coutts 2024	20	432	5	452	7.3%	4.19 [1.58, 11.05]	2024
Xiong 2024	35	264	33	252	24.1%	1.01 [0.65, 1.58]	2024
Chen 2024	1	50	1	49	1.0%	0.98 [0.06, 15.23]	2024
Albers 2024	43	228	39	230	27.7%	1.11 [0.75, 1.65]	2024
Cheng 2025	12	111	12	113	11.1%	1.02 [0.48, 2.17]	2025
Yogendrakumar 2025	11	120	8	122	8.7%	1.40 [0.58, 3.35]	2025
**Total (95% CI)**		**1533**		**1548**	**100.0%**	**1.25 [0.95, 1.65]**	
Total events	153		121				

Heterogeneity: Tau² = 0.03; Chi² = 8.92, df = 7 (P = 0.26); I² = 22%
Test for overall effect: Z = 1.57 (P = 0.12)

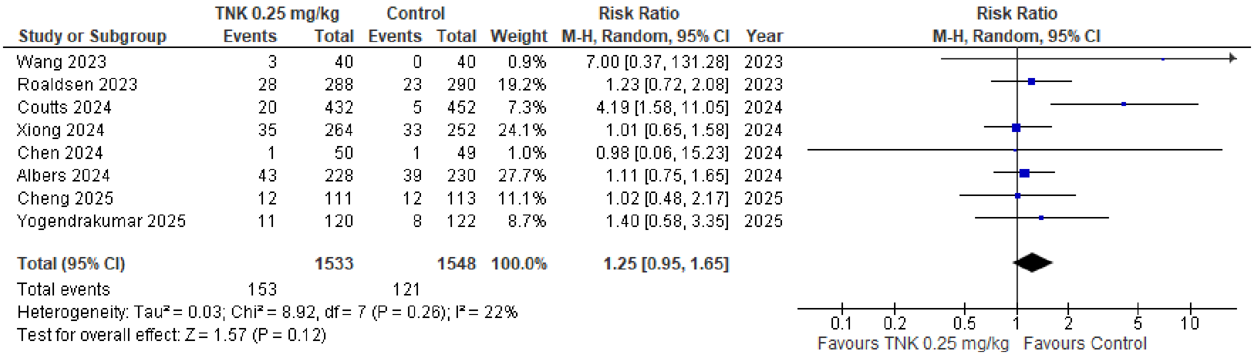

7. Symptomatic intracranial hemorrhage (sICH)

Study or Subgroup	TNK 0.25 mg/kg Events	TNK 0.25 mg/kg Total	Control Events	Control Total	Weight	Risk Ratio M-H, Random, 95% CI	Year
Roaldsen 2023	6	288	3	290	16.4%	2.01 [0.51, 7.98]	2023
Albers 2024	7	228	5	230	24.2%	1.41 [0.45, 4.38]	2024
Coutts 2024	8	432	2	452	13.1%	4.19 [0.89, 19.60]	2024
Xiong 2024	8	264	2	252	13.1%	3.82 [0.82, 17.81]	2024
Chen 2024	1	50	0	49	3.1%	2.94 [0.12, 70.50]	2024
Yogendrakumar 2025	5	120	1	122	6.8%	5.08 [0.60, 42.87]	2025
Cheng 2025	6	111	5	113	23.2%	1.22 [0.38, 3.89]	2025
**Total (95% CI)**		**1493**		**1508**	**100.0%**	**2.12 [1.21, 3.71]**	
Total events	41		18				

Heterogeneity: Tau² = 0.00; Chi² = 3.41, df = 6 (P = 0.76); I² = 0%
Test for overall effect: Z = 2.64 (P = 0.008)

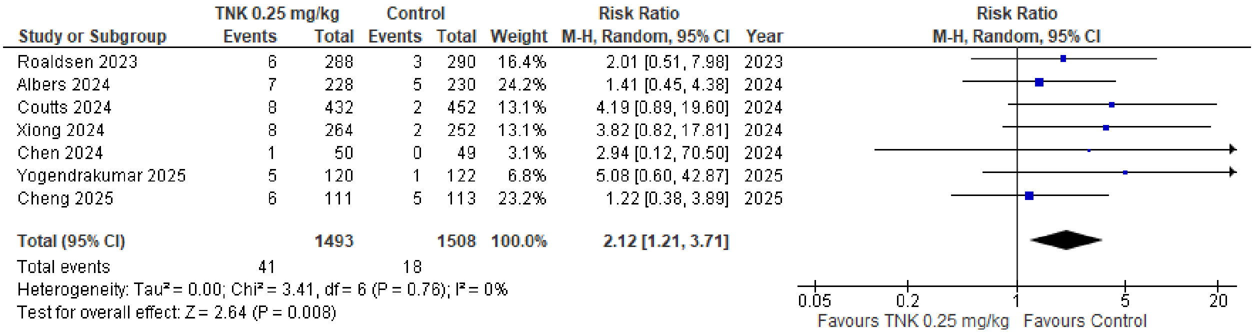

8. Poor functional outcomes (mRS 5-6 at 90 days)

Study or Subgroup	Experimental Events	Experimental Total	Control Events	Control Total	Weight	Risk Ratio M-H, Random, 95% CI	Year
Roaldsen 2023	34	288	31	290	16.2%	1.10 [0.70, 1.75]	2023
Wang 2023	4	40	0	40	0.6%	9.00 [0.50, 161.86]	2023
Coutts 2024	26	432	12	452	8.9%	2.27 [1.16, 4.44]	2024
Xiong 2024	46	264	52	252	22.5%	0.84 [0.59, 1.21]	2024
Albers 2024	59	226	57	229	26.0%	1.05 [0.77, 1.44]	2024
Chen 2024	3	50	2	49	1.5%	1.47 [0.26, 8.42]	2024
Yogendrakumar 2025	19	120	21	122	11.8%	0.92 [0.52, 1.62]	2025
Cheng 2025	22	111	20	113	12.5%	1.12 [0.65, 1.93]	2025
**Total (95% CI)**		**1531**		**1547**	**100.0%**	**1.09 [0.88, 1.36]**	
Total events	213		195				

Heterogeneity: Tau² = 0.02; Chi² = 9.13, df = 7 (P = 0.24); I² = 23%
Test for overall effect: Z = 0.77 (P = 0.44)

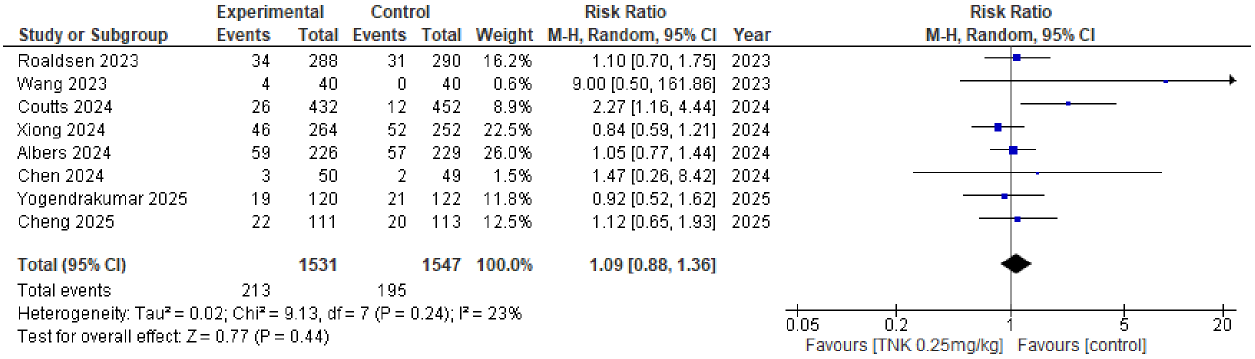

9. Any intracranial hemorrhage (ICH)

Study or Subgroup	TNK 0.25 mg/kg Events	TNK 0.25 mg/kg Total	Control Events	Control Total	Weight	Risk Ratio M-H, Random, 95% CI	Year
Roaldsen 2023	33	288	30	290	51.7%	1.11 [0.69, 1.77]	2023
Chen 2024	5	50	0	49	2.1%	10.78 [0.61, 189.95]	2024
Cheng 2025	27	111	21	113	46.3%	1.31 [0.79, 2.17]	2025
**Total (95% CI)**		**449**		**452**	**100.0%**	**1.25 [0.83, 1.90]**	
Total events	65		51				

Heterogeneity: Tau² = 0.03; Chi² = 2.51, df = 2 (P = 0.29); I² = 20%
Test for overall effect: Z = 1.07 (P = 0.28)

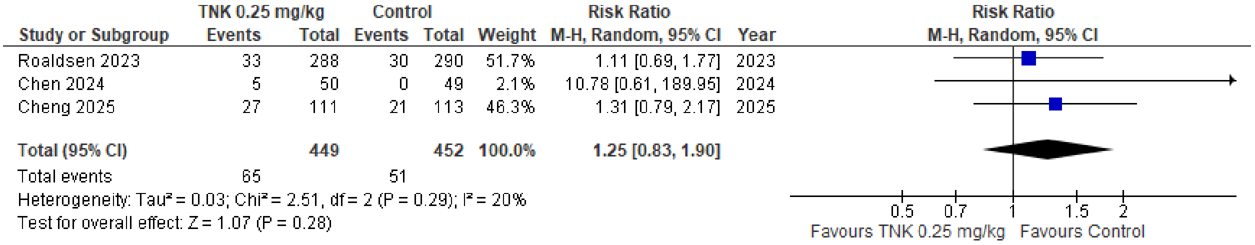

10. Systemic bleeding

Study or Subgroup	TNK 0.25 mg/kg Events	TNK 0.25 mg/kg Total	Control Events	Control Total	Weight	Risk Ratio M-H, Random, 95% CI	Year
Roaldsen 2023	1	288	0	290	7.7%	3.02 [0.12, 73.85]	2023
Xiong 2024	5	264	2	252	29.5%	2.39 [0.47, 12.19]	2024
Cheng 2025	5	111	7	113	62.8%	0.73 [0.24, 2.22]	2025
**Total (95% CI)**		**663**		**655**	**100.0%**	**1.15 [0.48, 2.79]**	
Total events	11		9				

Heterogeneity: Tau² = 0.00; Chi² = 1.78, df = 2 (P = 0.41); I² = 0%
Test for overall effect: Z = 0.31 (P = 0.75)

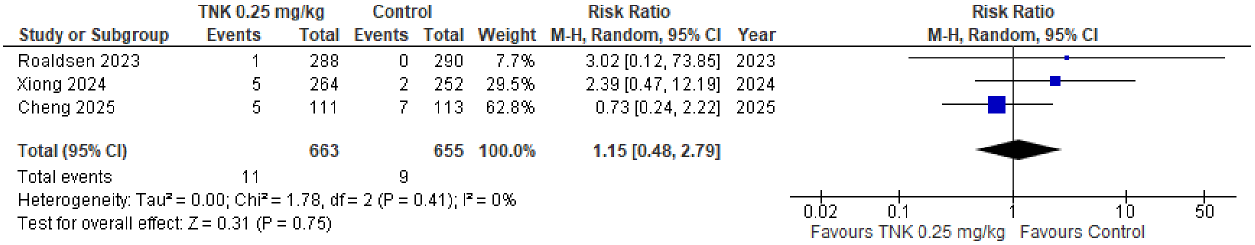

11. Parenchymal hematoma type 2 (PH-2)

Study or Subgroup	TNK 0.25 mg/kg Events	TNK 0.25 mg/kg Total	Control Events	Control Total	Weight	Risk Ratio M-H, Random, 95% CI	Year
Roaldsen 2023	7	280	5	290	23.7%	1.45 [0.47, 4.51]	2023
Wang 2023	1	40	0	40	3.0%	3.00 [0.13, 71.51]	2023
Albers 2024	8	228	6	230	28.1%	1.35 [0.47, 3.82]	2024
Yogendrakumar 2025	7	120	4	122	21.2%	1.78 [0.53, 5.92]	2025
Cheng 2025	10	111	4	113	24.0%	2.55 [0.82, 7.88]	2025
**Total (95% CI)**		**779**		**795**	**100.0%**	**1.73 [1.00, 3.02]**	
Total events	33		19				

Heterogeneity: Tau² = 0.00; Chi² = 0.88, df = 4 (P = 0.93); I² = 0%
Test for overall effect: Z = 1.95 (P = 0.05)

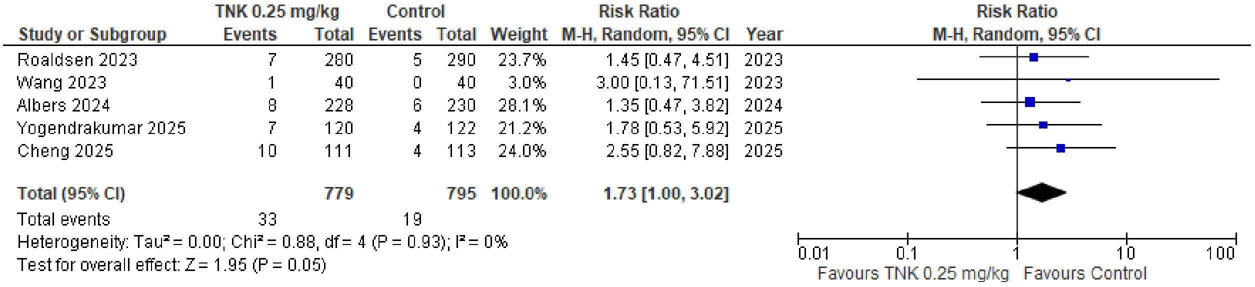

Subgroup analysis according to the inclusion criteria of mRS

1. Excellent functional outcomes (mRS 0-1 at 90 days)

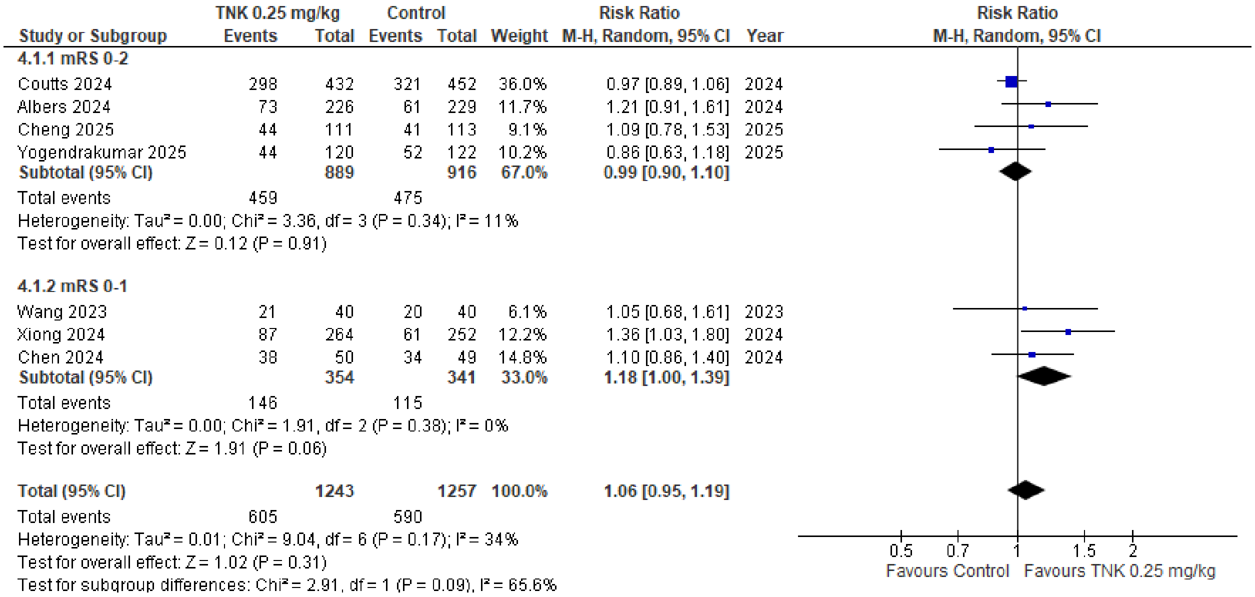

2. Favorable functional outcomes (mRS 0-2 at 90 days)

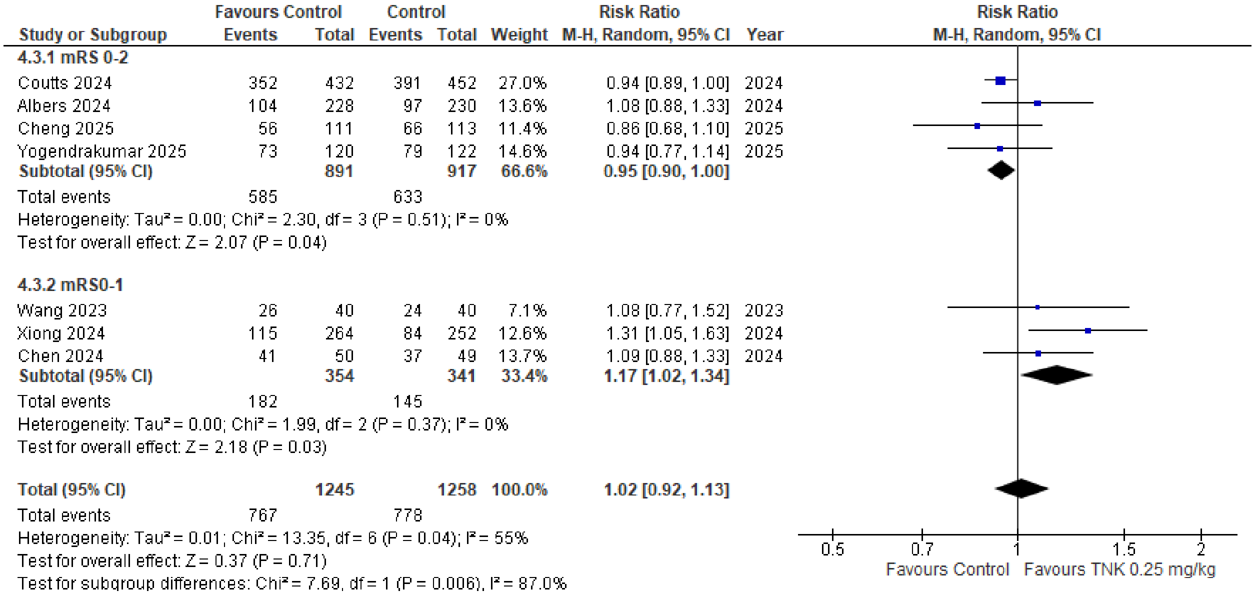

3. Major neurological improvements

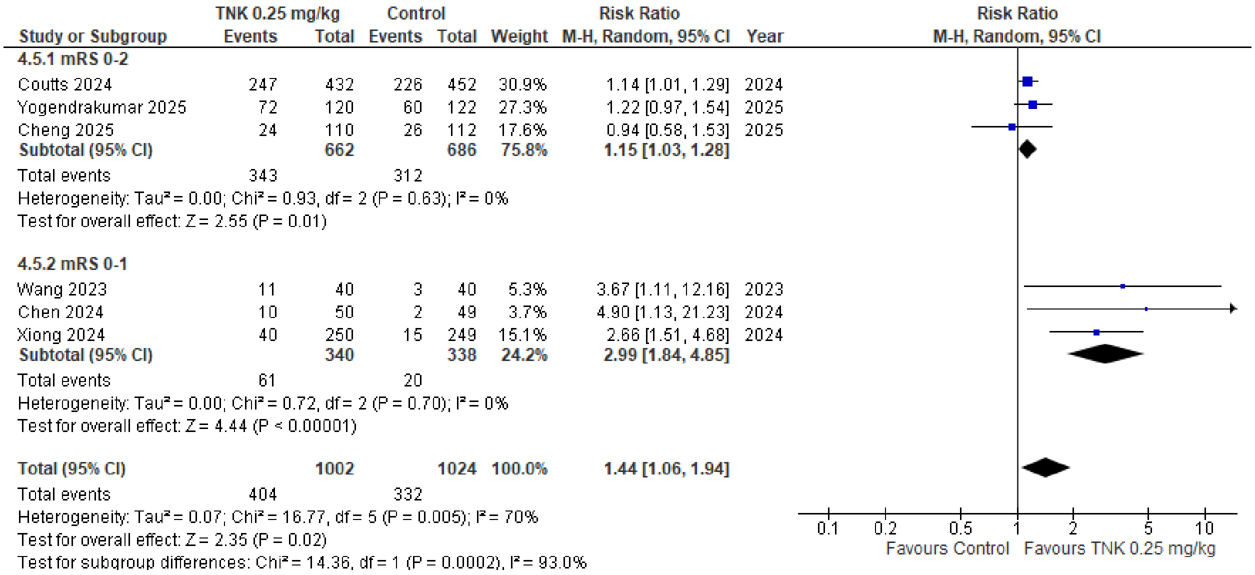

4. Death at 90 days

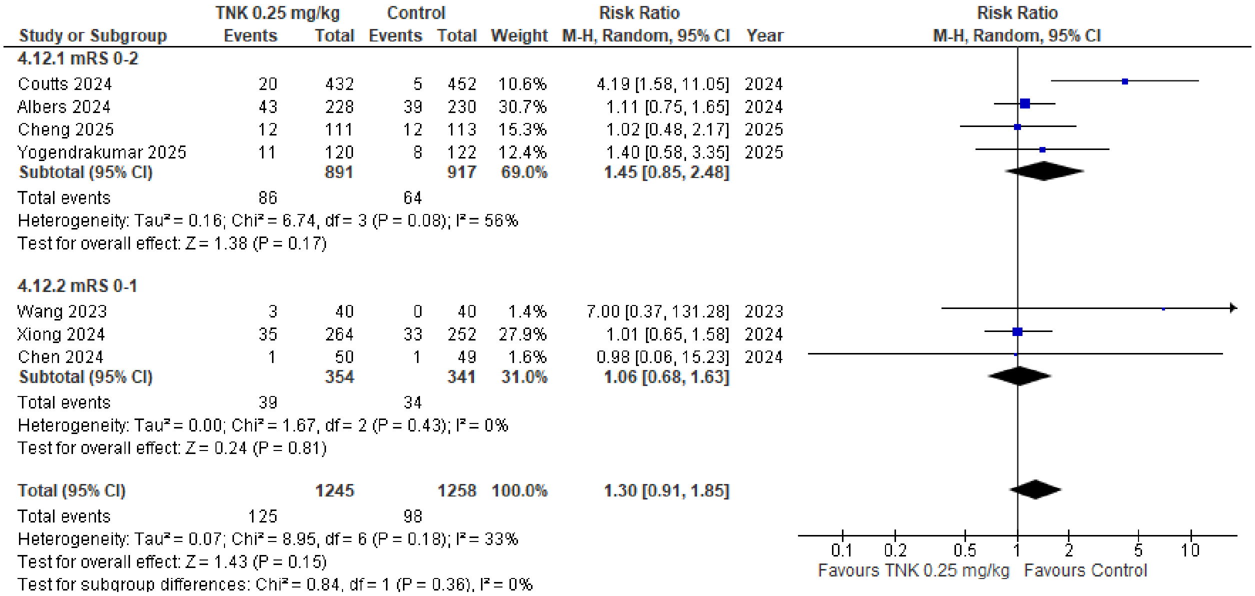

5. Symptomatic intracranial hemorrhage (sICH)

Study or Subgroup	TNK 0.25 mg/kg Events	Total	Control Events	Total	Weight	Risk Ratio M-H, Random, 95% CI	Year
**4.8.1 mRS 0-2**							
Albers 2024	7	228	5	230	29.0%	1.41 [0.45, 4.38]	2024
Coutts 2024	8	432	2	452	15.6%	4.19 [0.89, 19.60]	2024
Yogendrakumar 2025	5	120	1	122	8.2%	5.08 [0.60, 42.87]	2025
Cheng 2025	6	111	5	113	27.8%	1.22 [0.38, 3.89]	2025
**Subtotal (95% CI)**		**891**		**917**	**80.6%**	**1.89 [0.96, 3.73]**	
Total events	26		13				
Heterogeneity: Tau² = 0.00; Chi² = 2.70, df = 3 (P = 0.44); I² = 0%							
Test for overall effect: Z = 1.83 (P = 0.07)							
**4.8.2 mRS 0-1**							
Xiong 2024	8	264	2	252	15.7%	3.82 [0.82, 17.81]	2024
Chen 2024	1	50	0	49	3.7%	2.94 [0.12, 70.50]	2024
**Subtotal (95% CI)**		**314**		**301**	**19.4%**	**3.63 [0.91, 14.52]**	
Total events	9		2				
Heterogeneity: Tau² = 0.00; Chi² = 0.02, df = 1 (P = 0.88); I² = 0%							
Test for overall effect: Z = 1.82 (P = 0.07)							
**Total (95% CI)**		**1205**		**1218**	**100.0%**	**2.14 [1.16, 3.95]**	
Total events	35		15				
Heterogeneity: Tau² = 0.00; Chi² = 3.41, df = 5 (P = 0.64); I² = 0%							
Test for overall effect: Z = 2.45 (P = 0.01)							
Test for subgroup differences: Chi² = 0.69, df = 1 (P = 0.41), I² = 0%							

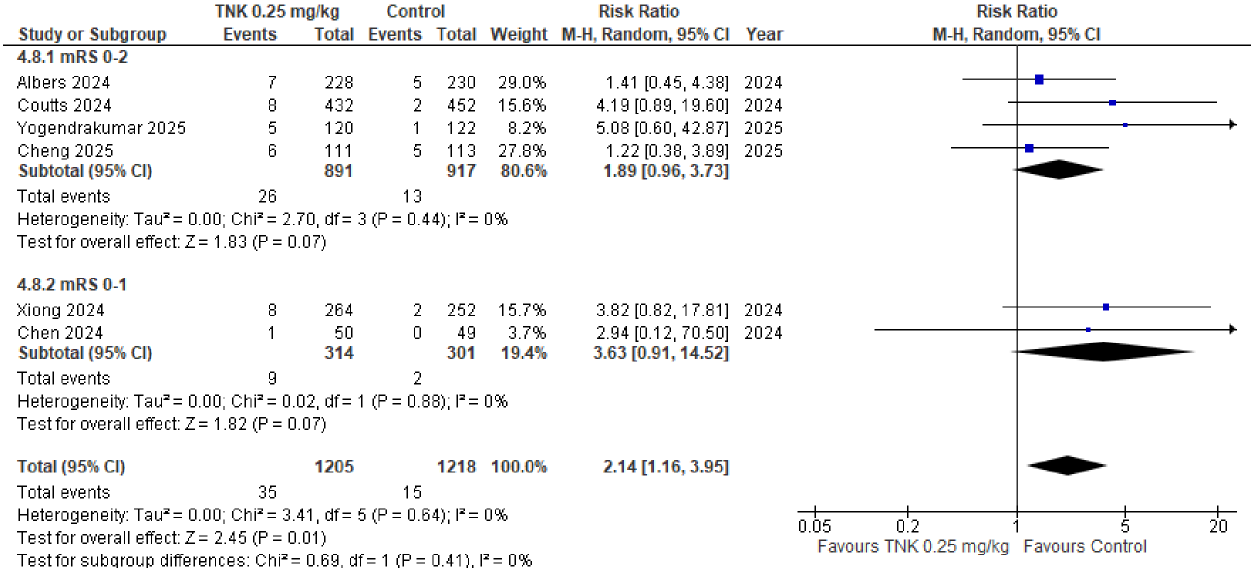

6. Poor functional outcomes (mRS 5-6 at 90 days)

Study or Subgroup	Experimental Events	Total	Control Events	Total	Weight	Risk Ratio M-H, Random, 95% CI	Year
**4.2.1 mRS 0-2**							
Albers 2024	59	226	57	229	28.4%	1.05 [0.77, 1.44]	2024
Coutts 2024	26	432	12	452	11.9%	2.27 [1.16, 4.44]	2024
Cheng 2025	22	111	20	113	15.9%	1.12 [0.65, 1.93]	2025
Yogendrakumar 2025	19	120	21	122	15.1%	0.92 [0.52, 1.62]	2025
**Subtotal (95% CI)**		**889**		**916**	**71.4%**	**1.18 [0.86, 1.62]**	
Total events	126		110				
Heterogeneity: Tau² = 0.04; Chi² = 4.91, df = 3 (P = 0.18); I² = 39%							
Test for overall effect: Z = 1.00 (P = 0.32)							
**4.2.2 mRS 0-1**							
Wang 2023	4	40	0	40	0.8%	9.00 [0.50, 161.86]	2023
Chen 2024	3	50	2	49	2.3%	1.47 [0.26, 8.42]	2024
Xiong 2024	46	264	52	252	25.5%	0.84 [0.59, 1.21]	2024
**Subtotal (95% CI)**		**354**		**341**	**28.6%**	**1.18 [0.46, 2.98]**	
Total events	53		54				
Heterogeneity: Tau² = 0.29; Chi² = 2.95, df = 2 (P = 0.23); I² = 32%							
Test for overall effect: Z = 0.34 (P = 0.73)							
**Total (95% CI)**		**1243**		**1257**	**100.0%**	**1.11 [0.85, 1.45]**	
Total events	179		164				
Heterogeneity: Tau² = 0.04; Chi² = 9.12, df = 6 (P = 0.17); I² = 34%							
Test for overall effect: Z = 0.73 (P = 0.46)							
Test for subgroup differences: Chi² = 0.00, df = 1 (P = 1.00), I² = 0%							

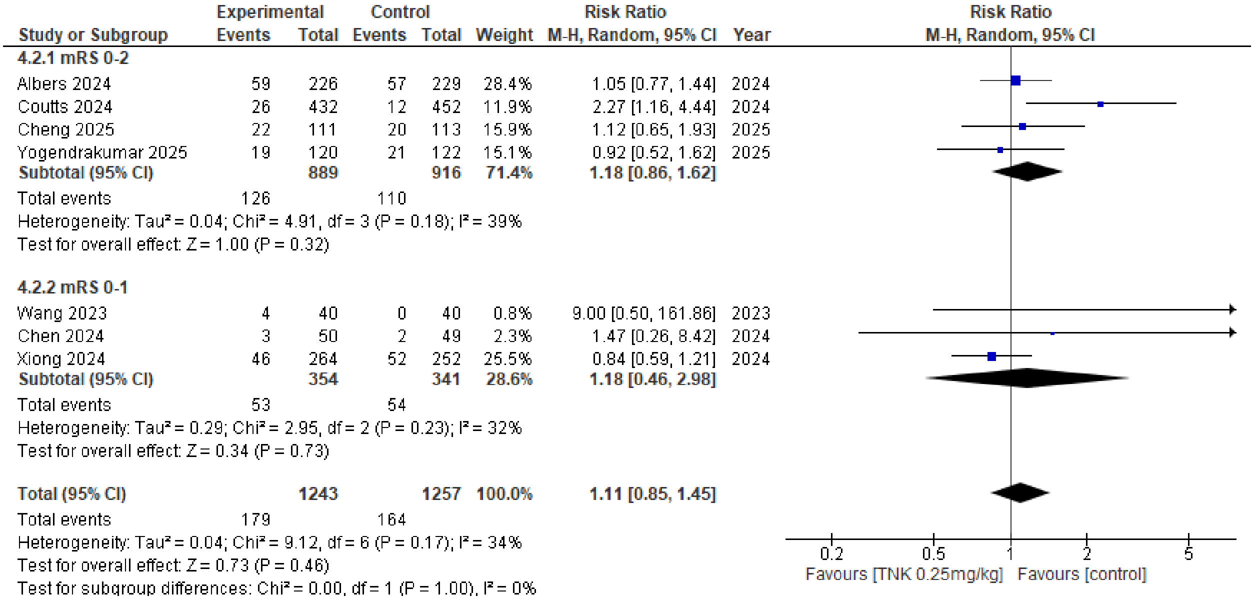

comparison between TNK 0.25 mg/kg vs best medical management

1. Excellent functional outcome (mRS 0-1 at 90 days)

Study or Subgroup	TNK 0.25 mg/kg Events	TNK 0.25 mg/kg Total	Control Events	Control Total	Weight	Risk Ratio M-H, Random, 95% CI	Year
Roaldsen 2023	130	288	111	290	21.2%	1.18 [0.97, 1.43]	2023
Wang 2023	21	40	20	40	7.7%	1.05 [0.68, 1.61]	2023
Coutts 2024	298	432	321	452	33.5%	0.97 [0.89, 1.06]	2024
Xiong 2024	87	264	61	252	14.3%	1.36 [1.03, 1.80]	2024
Cheng 2025	44	111	41	113	11.1%	1.09 [0.78, 1.53]	2025
Yogendrakumar 2025	44	120	52	122	12.2%	0.86 [0.63, 1.18]	2025
**Total (95% CI)**		**1255**		**1269**	**100.0%**	**1.07 [0.93, 1.22]**	
Total events	624		606				

Heterogeneity: Tau² = 0.01; Chi² = 9.54, df = 5 (P = 0.09); I² = 48%
Test for overall effect: Z = 0.96 (P = 0.34)

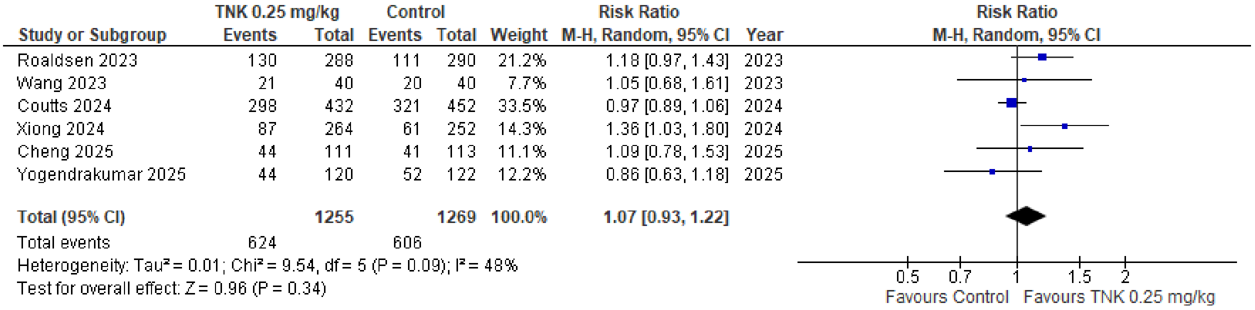

2. Favorable functional outcomes (mRS 0-2 at 90 days)

Study or Subgroup	TNK 0.25 mg/kg Events	TNK 0.25 mg/kg Total	Control Events	Control Total	Weight	Risk Ratio M-H, Random, 95% CI	Year
Wang 2023	26	40	24	40	7.2%	1.08 [0.77, 1.52]	2023
Roaldsen 2023	177	288	173	290	21.8%	1.03 [0.90, 1.18]	2023
Xiong 2024	115	264	84	252	13.2%	1.31 [1.05, 1.63]	2024
Coutts 2024	352	432	391	452	30.5%	0.94 [0.89, 1.00]	2024
Yogendrakumar 2025	73	120	79	122	15.4%	0.94 [0.77, 1.14]	2025
Cheng 2025	56	111	66	113	11.9%	0.86 [0.68, 1.10]	2025
**Total (95% CI)**		**1255**		**1269**	**100.0%**	**1.00 [0.90, 1.11]**	
Total events	799		817				

Heterogeneity: Tau² = 0.01; Chi² = 11.61, df = 5 (P = 0.04); I² = 57%
Test for overall effect: Z = 0.04 (P = 0.97)

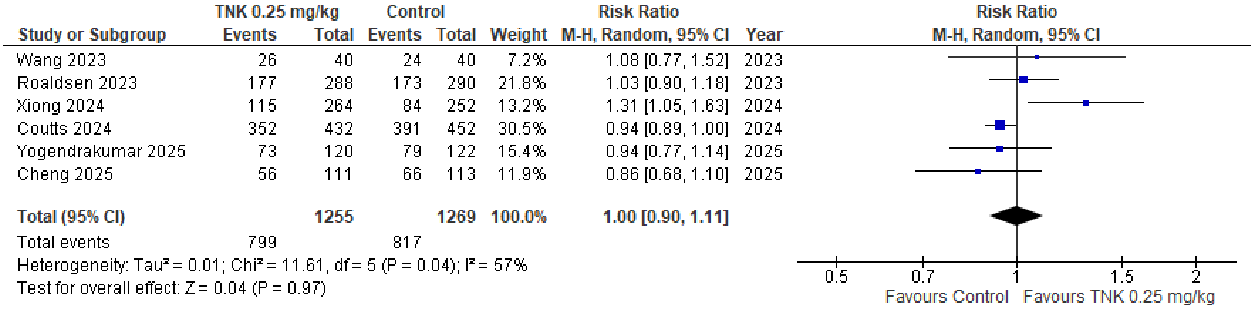

3. Major neurological improvement

Study or Subgroup	TNK 0.25 mg/kg Events	TNK 0.25 mg/kg Total	Control Events	Control Total	Weight	Risk Ratio M-H, Random, 95% CI	Year
Wang 2023	11	40	3	40	4.7%	3.67 [1.11, 12.16]	2023
Xiong 2024	40	250	15	249	14.6%	2.66 [1.51, 4.68]	2024
Coutts 2024	247	432	226	452	34.1%	1.14 [1.01, 1.29]	2024
Cheng 2025	24	110	26	112	17.3%	0.94 [0.58, 1.53]	2025
Yogendrakumar 2025	72	120	60	122	29.2%	1.22 [0.97, 1.54]	2025
**Total (95% CI)**		**952**		**975**	**100.0%**	**1.35 [1.02, 1.78]**	
Total events	394		330				

Heterogeneity: Tau² = 0.06; Chi² = 12.88, df = 4 (P = 0.01); I² = 69%
Test for overall effect: Z = 2.08 (P = 0.04)

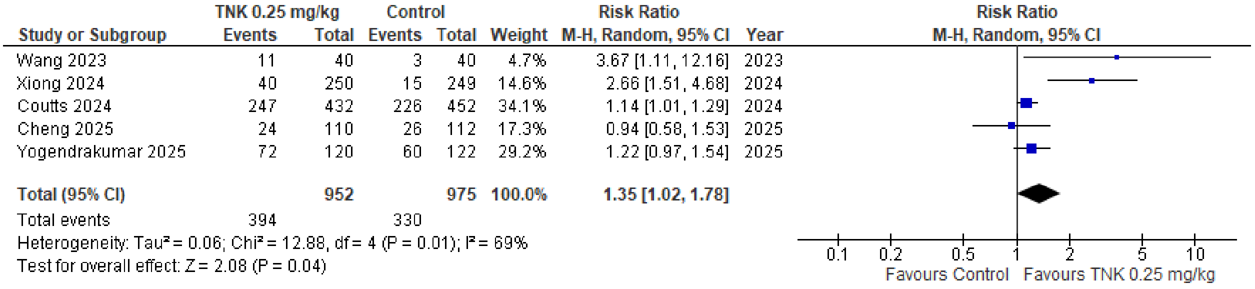

4. Death at 90 days

Study or Subgroup	TNK 0.25 mg/kg Events	TNK 0.25 mg/kg Total	Control Events	Control Total	Weight	Risk Ratio M-H, Random, 95% CI	Year
Roaldsen 2023	28	288	23	290	25.1%	1.23 [0.72, 2.08]	2023
Wang 2023	3	40	0	40	1.9%	7.00 [0.37, 131.28]	2023
Coutts 2024	20	432	5	452	12.5%	4.19 [1.58, 11.05]	2024
Xiong 2024	35	264	33	252	28.6%	1.01 [0.65, 1.58]	2024
Cheng 2025	12	111	12	113	17.4%	1.02 [0.48, 2.17]	2025
Yogendrakumar 2025	11	120	8	122	14.5%	1.40 [0.58, 3.35]	2025
**Total (95% CI)**		**1255**		**1269**	**100.0%**	**1.38 [0.92, 2.08]**	
Total events	109		81				

Heterogeneity: Tau² = 0.10; Chi² = 8.65, df = 5 (P = 0.12); I² = 42%
Test for overall effect: Z = 1.55 (P = 0.12)

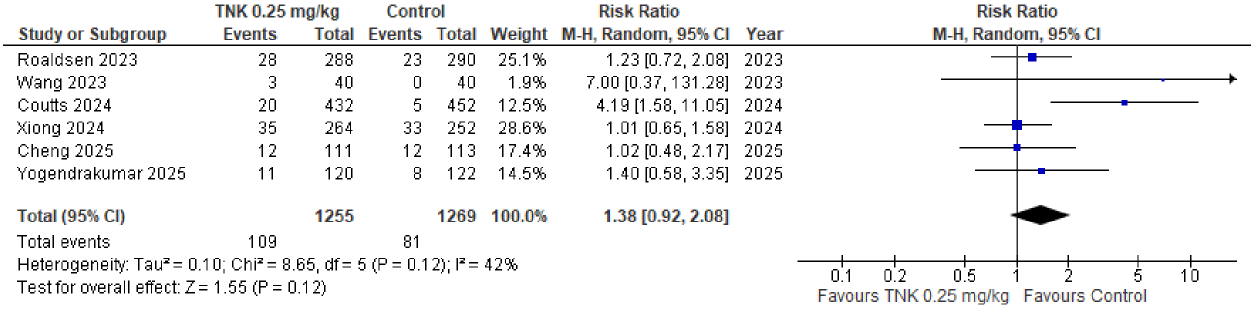

5. Symptomatic intracranial hemorrhage (sICH)

Study or Subgroup	TNK 0.25 mg/kg Events	TNK 0.25 mg/kg Total	Control Events	Control Total	Weight	Risk Ratio M-H, Random, 95% CI	Year
Roaldsen 2023	6	288	3	290	22.6%	2.01 [0.51, 7.98]	2023
Coutts 2024	8	432	2	452	18.0%	4.19 [0.89, 19.60]	2024
Xiong 2024	8	264	2	252	18.1%	3.82 [0.82, 17.81]	2024
Yogendrakumar 2025	5	120	1	122	9.4%	5.08 [0.60, 42.87]	2025
Cheng 2025	6	111	5	113	32.0%	1.22 [0.38, 3.89]	2025
**Total (95% CI)**		**1215**		**1229**	**100.0%**	**2.40 [1.25, 4.61]**	
Total events	33		13				

Heterogeneity: Tau² = 0.00; Chi² = 2.73, df = 4 (P = 0.60); I² = 0%
Test for overall effect: Z = 2.62 (P = 0.009)

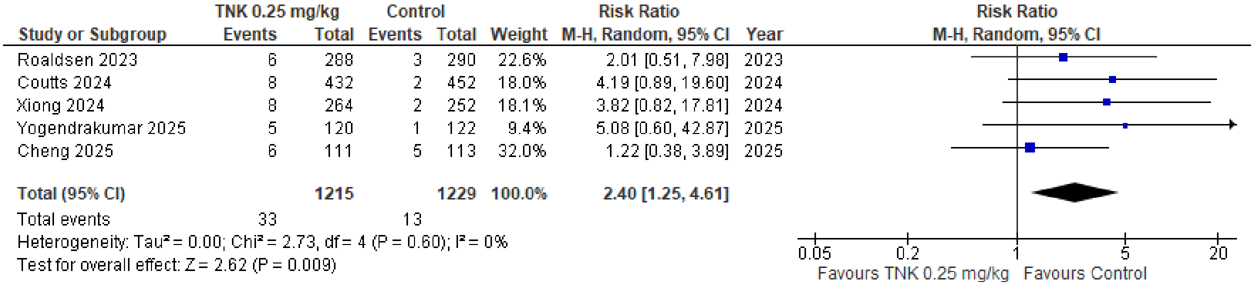

6. Poor functional outcome (mRS 5-6 at 90 days)

Study or Subgroup	Experimental Events	Experimental Total	Control Events	Control Total	Weight	Risk Ratio M-H, Random, 95% CI	Year
Roaldsen 2023	34	288	31	290	21.9%	1.10 [0.70, 1.75]	2023
Wang 2023	4	40	0	40	1.2%	9.00 [0.50, 161.86]	2023
Xiong 2024	46	264	52	252	26.7%	0.84 [0.59, 1.21]	2024
Coutts 2024	26	432	12	452	14.3%	2.27 [1.16, 4.44]	2024
Yogendrakumar 2025	19	120	21	122	17.6%	0.92 [0.52, 1.62]	2025
Cheng 2025	22	111	20	113	18.3%	1.12 [0.65, 1.93]	2025
**Total (95% CI)**		**1255**		**1269**	**100.0%**	**1.13 [0.83, 1.56]**	
Total events	151		136				

Heterogeneity: Tau² = 0.06; Chi² = 9.01, df = 5 (P = 0.11); I² = 45%
Test for overall effect: Z = 0.78 (P = 0.44)

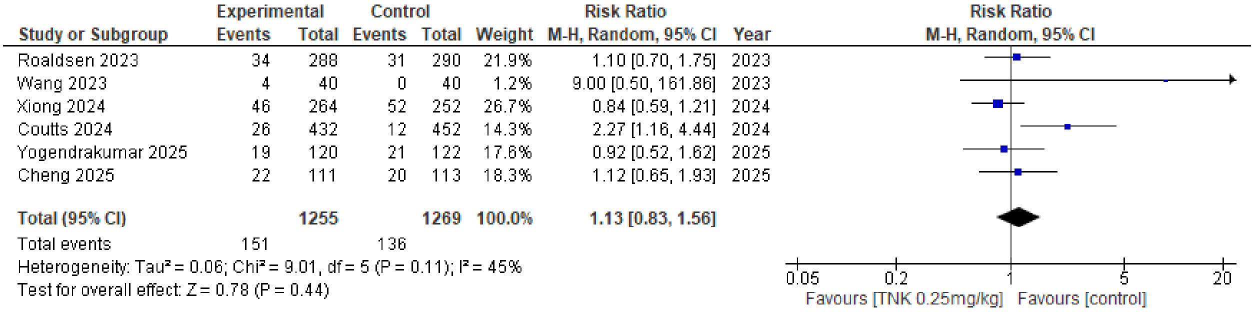

7. Systemic bleeding

Study or Subgroup	TNK 0.25 mg/kg Events	TNK 0.25 mg/kg Total	Control Events	Control Total	Weight	Risk Ratio M-H, Random, 95% CI	Year
Roaldsen 2023	1	288	0	290	7.7%	3.02 [0.12, 73.85]	2023
Xiong 2024	5	264	2	252	29.5%	2.39 [0.47, 12.19]	2024
Cheng 2025	5	111	7	113	62.8%	0.73 [0.24, 2.22]	2025
**Total (95% CI)**		**663**		**655**	**100.0%**	**1.15 [0.48, 2.79]**	
Total events	11		9				

Heterogeneity: Tau² = 0.00; Chi² = 1.78, df = 2 (P = 0.41); I² = 0%
Test for overall effect: Z = 0.31 (P = 0.75)

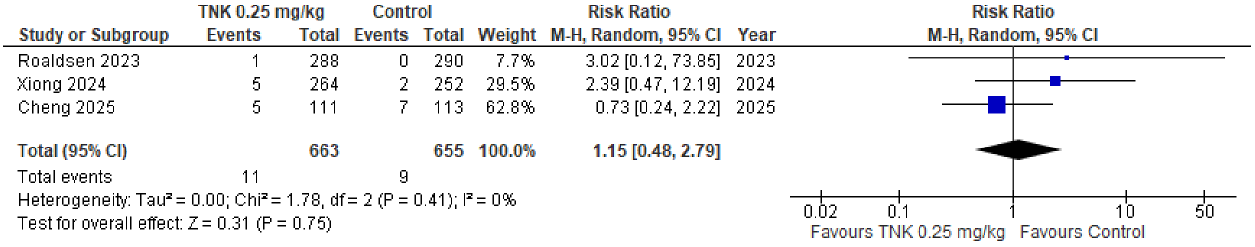

8. Parenchymal hematoma type 2 (PH-2)

Study or Subgroup	TNK 0.25 mg/kg Events	Total	Control Events	Total	Weight	Risk Ratio M-H, Random, 95% CI	Year
Roaldsen 2023	7	280	5	290	33.0%	1.45 [0.47, 4.51]	2023
Wang 2023	1	40	0	40	4.2%	3.00 [0.13, 71.51]	2023
Cheng 2025	10	111	4	113	33.4%	2.55 [0.82, 7.88]	2025
Yogendrakumar 2025	7	120	4	122	29.4%	1.78 [0.53, 5.92]	2025
**Total (95% CI)**		**551**		**565**	**100.0%**	**1.92 [1.00, 3.68]**	
Total events	25		13				

Heterogeneity: Tau² = 0.00; Chi² = 0.57, df = 3 (P = 0.90); I² = 0%
Test for overall effect: Z = 1.95 (P = 0.05)

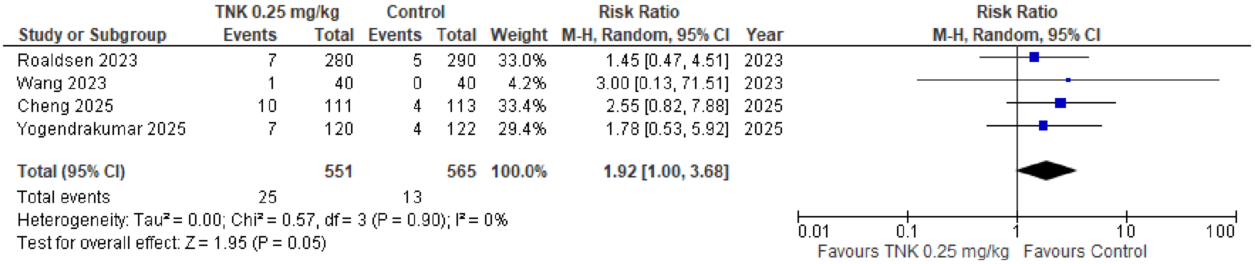

Subgroup analysis according to the allowance of post-procedure mechanical thrombectomy (MT)

1. Excellent functional outcomes (mRS 0-1)

Study or Subgroup	TNK 0.25 mg/kg Events	Total	Control Events	Total	Weight	Risk Ratio M-H, Random, 95% CI	Year
**6.1.1 MT not allowed**							
Wang 2023	21	40	20	40	5.1%	1.05 [0.68, 1.61]	2023
Xiong 2024	87	264	61	252	10.2%	1.36 [1.03, 1.80]	2024
Chen 2024	38	50	34	49	12.4%	1.10 [0.86, 1.40]	2024
**Subtotal (95% CI)**		**354**		**341**	**27.8%**	**1.18 [1.00, 1.39]**	
Total events	146		115				
Heterogeneity: Tau² = 0.00; Chi² = 1.91, df = 2 (P = 0.38); I² = 0%							
Test for overall effect: Z = 1.91 (P = 0.06)							
**6.1.2 MT allowed**							
Roaldsen 2023	130	288	111	290	16.3%	1.18 [0.97, 1.43]	2023
Albers 2024	73	228	61	230	9.8%	1.21 [0.91, 1.61]	2024
Coutts 2024	298	432	321	452	29.7%	0.97 [0.89, 1.06]	2024
Cheng 2025	44	111	41	113	7.7%	1.09 [0.78, 1.53]	2025
Yogendrakumar 2025	44	120	52	122	8.6%	0.86 [0.63, 1.18]	2025
**Subtotal (95% CI)**		**1179**		**1207**	**72.2%**	**1.04 [0.93, 1.17]**	
Total events	589		586				
Heterogeneity: Tau² = 0.01; Chi² = 6.36, df = 4 (P = 0.17); I² = 37%							
Test for overall effect: Z = 0.68 (P = 0.50)							
**Total (95% CI)**		**1533**		**1548**	**100.0%**	**1.08 [0.97, 1.20]**	
Total events	735		701				

Heterogeneity: Tau² = 0.01; Chi² = 11.14, df = 7 (P = 0.13); I² = 37%
Test for overall effect: Z = 1.43 (P = 0.15)
Test for subgroup differences: Chi² = 1.37, df = 1 (P = 0.24), I² = 27.2%

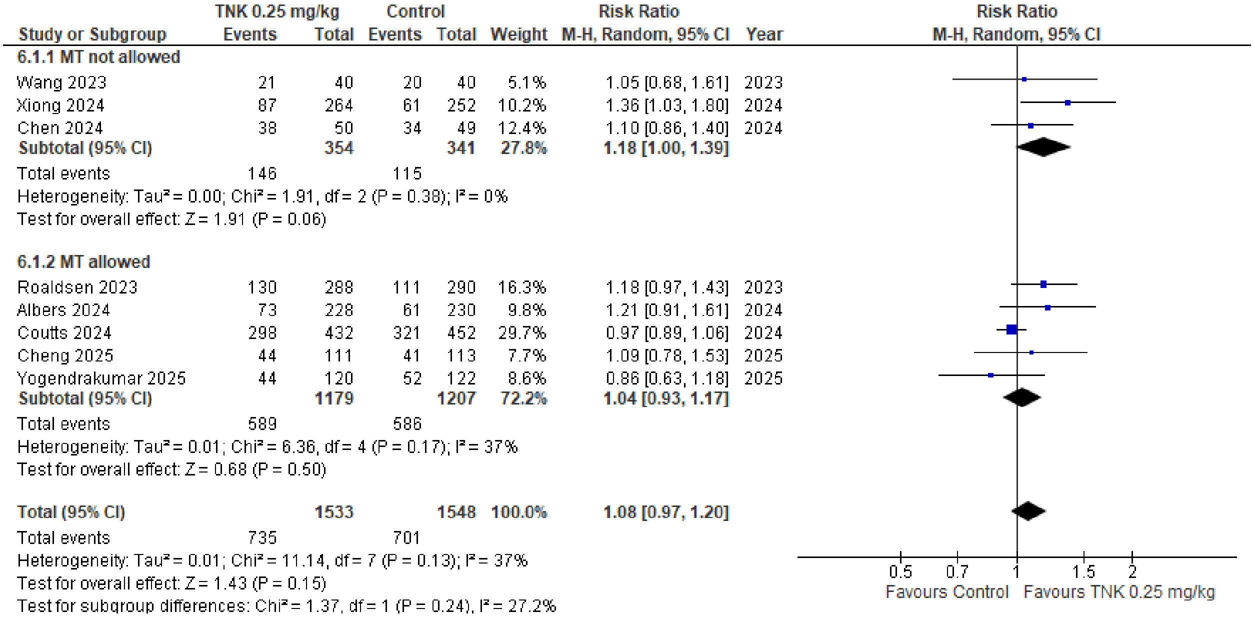

2. Favorable functional outcomes (mRS 0-2)

Study or Subgroup	Favours Control Events	Favours Control Total	Control Events	Control Total	Weight	Risk Ratio M-H, Random, 95% CI	Year
**6.3.1 MT not allowed**							
Wang 2023	26	40	24	40	5.2%	1.08 [0.77, 1.52]	2023
Xiong 2024	115	264	84	252	9.8%	1.31 [1.05, 1.63]	2024
Chen 2024	41	50	37	49	10.9%	1.09 [0.88, 1.33]	2024
**Subtotal (95% CI)**		**354**		**341**	**25.9%**	**1.17 [1.02, 1.34]**	
Total events	182		145				
Heterogeneity: Tau² = 0.00; Chi² = 1.99, df = 2 (P = 0.37); I² = 0%							
Test for overall effect: Z = 2.18 (P = 0.03)							
**6.3.2 MT allowed**							
Roaldsen 2023	177	288	173	290	17.2%	1.03 [0.90, 1.18]	2023
Coutts 2024	352	432	391	452	25.6%	0.94 [0.89, 1.00]	2024
Albers 2024	104	228	97	230	10.8%	1.08 [0.88, 1.33]	2024
Yogendrakumar 2025	73	120	79	122	11.6%	0.94 [0.77, 1.14]	2025
Cheng 2025	56	111	66	113	8.8%	0.86 [0.68, 1.10]	2025
**Subtotal (95% CI)**		**1179**		**1207**	**74.1%**	**0.96 [0.91, 1.00]**	
Total events	762		806				
Heterogeneity: Tau² = 0.00; Chi² = 3.91, df = 4 (P = 0.42); I² = 0%							
Test for overall effect: Z = 1.76 (P = 0.08)							
**Total (95% CI)**		**1533**		**1548**	**100.0%**	**1.02 [0.93, 1.11]**	
Total events	944		951				
Heterogeneity: Tau² = 0.01; Chi² = 13.98, df = 7 (P = 0.05); I² = 50%							
Test for overall effect: Z = 0.40 (P = 0.69)							
Test for subgroup differences: Chi² = 6.98, df = 1 (P = 0.008), I² = 85.7%							

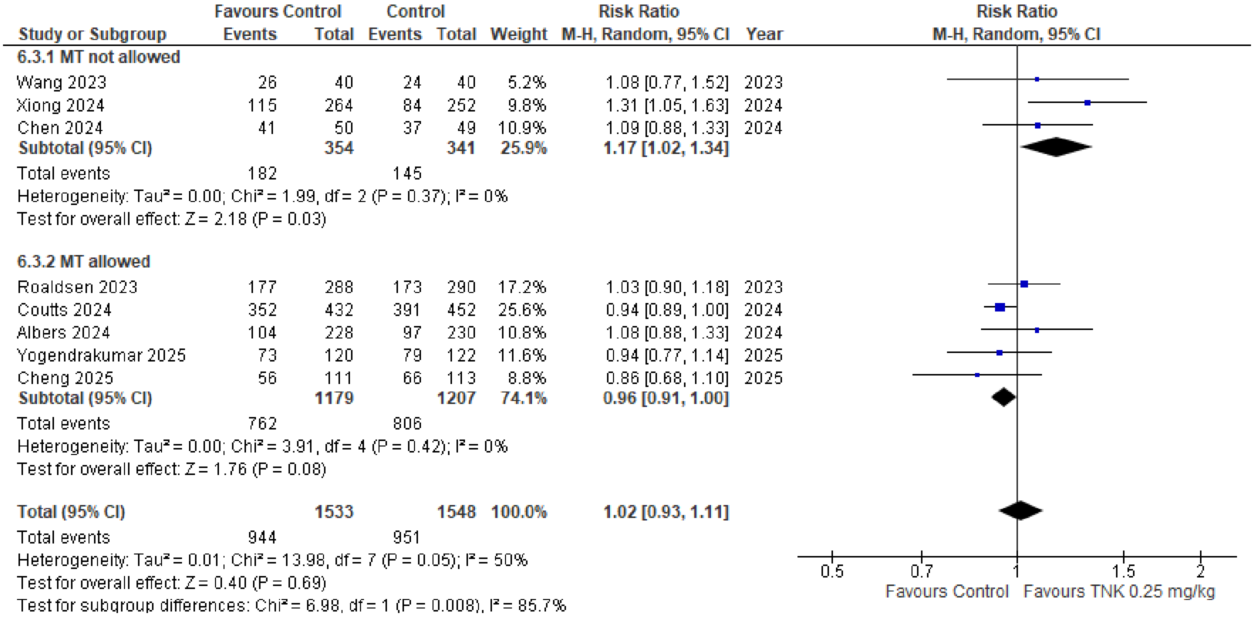

3. Major neurological improvements

Study or Subgroup	TNK 0.25 mg/kg Events	TNK 0.25 mg/kg Total	Control Events	Control Total	Weight	Risk Ratio M-H, Random, 95% CI	Year
**6.5.1 MT not allowed**							
Wang 2023	11	40	3	40	5.3%	3.67 [1.11, 12.16]	2023
Xiong 2024	40	250	15	249	15.1%	2.66 [1.51, 4.68]	2024
Chen 2024	10	50	2	49	3.7%	4.90 [1.13, 21.23]	2024
**Subtotal (95% CI)**		**340**		**338**	**24.2%**	**2.99 [1.84, 4.85]**	
Total events	61		20				
Heterogeneity: Tau² = 0.00; Chi² = 0.72, df = 2 (P = 0.70); I² = 0%							
Test for overall effect: Z = 4.44 (P < 0.00001)							
**6.5.2 MT allowed**							
Coutts 2024	247	432	226	452	30.9%	1.14 [1.01, 1.29]	2024
Yogendrakumar 2025	72	120	60	122	27.3%	1.22 [0.97, 1.54]	2025
Cheng 2025	24	110	26	112	17.6%	0.94 [0.58, 1.53]	2025
**Subtotal (95% CI)**		**662**		**686**	**75.8%**	**1.15 [1.03, 1.28]**	
Total events	343		312				
Heterogeneity: Tau² = 0.00; Chi² = 0.93, df = 2 (P = 0.63); I² = 0%							
Test for overall effect: Z = 2.55 (P = 0.01)							
**Total (95% CI)**		**1002**		**1024**	**100.0%**	**1.44 [1.06, 1.94]**	
Total events	404		332				
Heterogeneity: Tau² = 0.07; Chi² = 16.77, df = 5 (P = 0.005); I² = 70%							
Test for overall effect: Z = 2.35 (P = 0.02)							
Test for subgroup differences: Chi² = 14.36, df = 1 (P = 0.0002), I² = 93.0%							

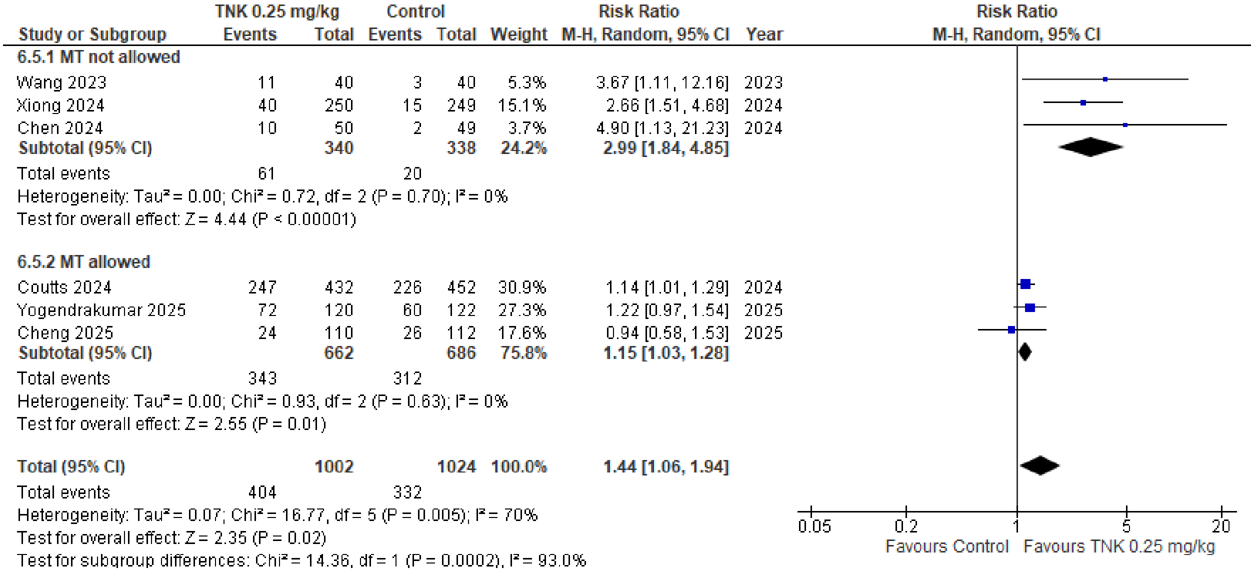

4. Death

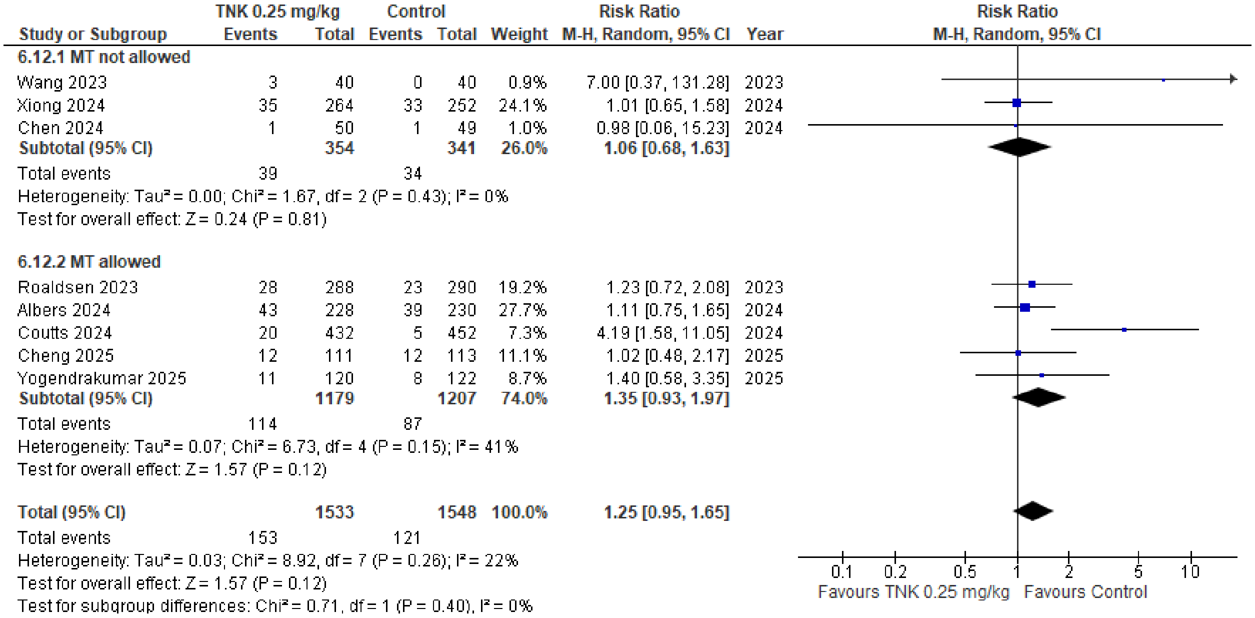

5. Symptomatic intracranial hemorrhage (sICH)

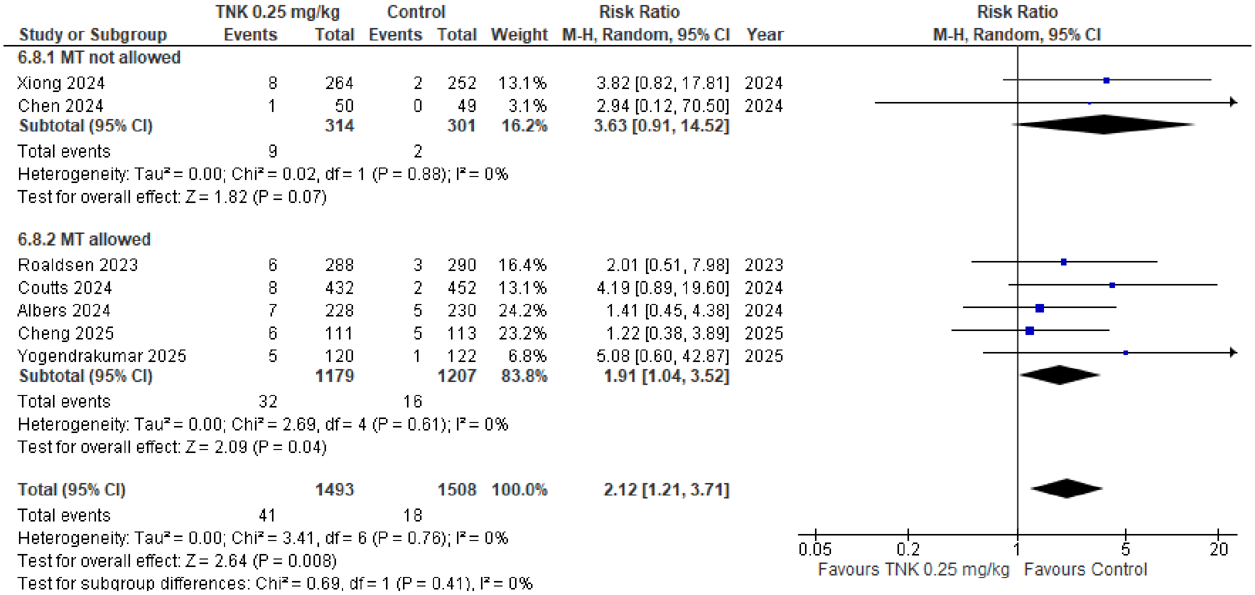

6. Poor functional outcomes (mRS 5-6)

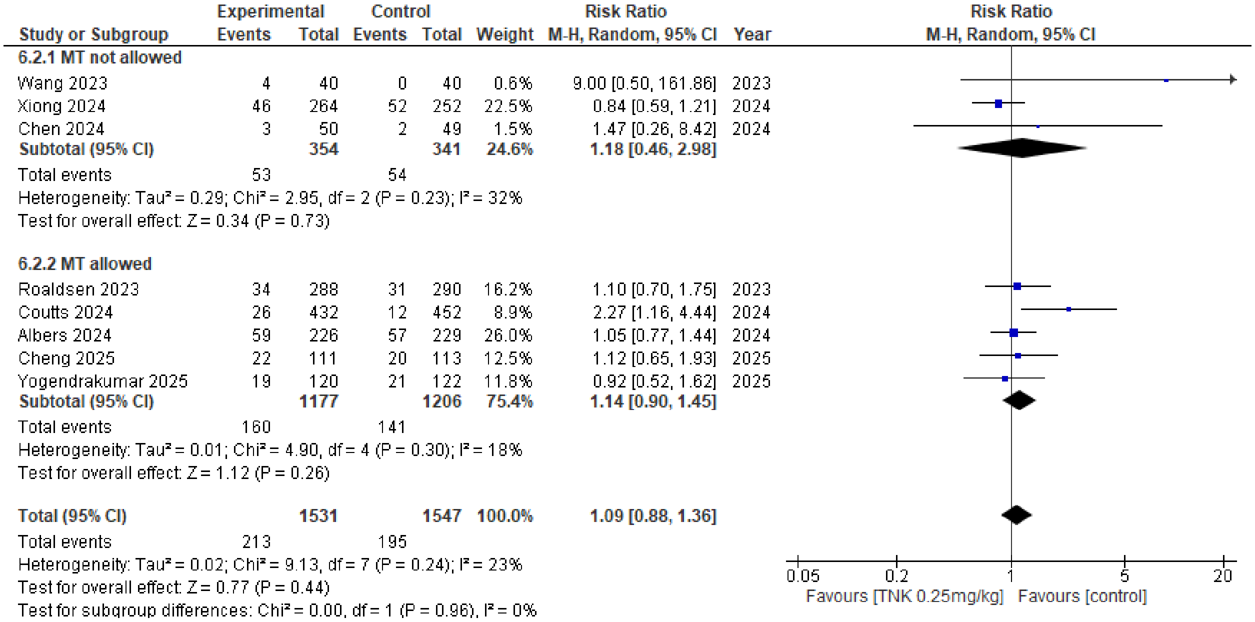

Study or Subgroup	Experimental Events	Experimental Total	Control Events	Control Total	Weight	Risk Ratio M-H, Random, 95% CI	Year
**6.2.1 MT not allowed**							
Wang 2023	4	40	0	40	0.6%	9.00 [0.50, 161.86]	2023
Xiong 2024	46	264	52	252	22.5%	0.84 [0.59, 1.21]	2024
Chen 2024	3	50	2	49	1.5%	1.47 [0.26, 8.42]	2024
**Subtotal (95% CI)**		**354**		**341**	**24.6%**	**1.18 [0.46, 2.98]**	
Total events	53		54				
Heterogeneity: Tau² = 0.29; Chi² = 2.95, df = 2 (P = 0.23); I² = 32%							
Test for overall effect: Z = 0.34 (P = 0.73)							
**6.2.2 MT allowed**							
Roaldsen 2023	34	288	31	290	16.2%	1.10 [0.70, 1.75]	2023
Coutts 2024	26	432	12	452	8.9%	2.27 [1.16, 4.44]	2024
Albers 2024	59	226	57	229	26.0%	1.05 [0.77, 1.44]	2024
Cheng 2025	22	111	20	113	12.5%	1.12 [0.65, 1.93]	2025
Yogendrakumar 2025	19	120	21	122	11.8%	0.92 [0.52, 1.62]	2025
**Subtotal (95% CI)**		**1177**		**1206**	**75.4%**	**1.14 [0.90, 1.45]**	
Total events	160		141				
Heterogeneity: Tau² = 0.01; Chi² = 4.90, df = 4 (P = 0.30); I² = 18%							
Test for overall effect: Z = 1.12 (P = 0.26)							
**Total (95% CI)**		**1531**		**1547**	**100.0%**	**1.09 [0.88, 1.36]**	
Total events	213		195				
Heterogeneity: Tau² = 0.02; Chi² = 9.13, df = 7 (P = 0.24); I² = 23%							
Test for overall effect: Z = 0.77 (P = 0.44)							
Test for subgroup differences: Chi² = 0.00, df = 1 (P = 0.96), I² = 0%							

mRS distribution at 90 days

1. Roaldsen 2023

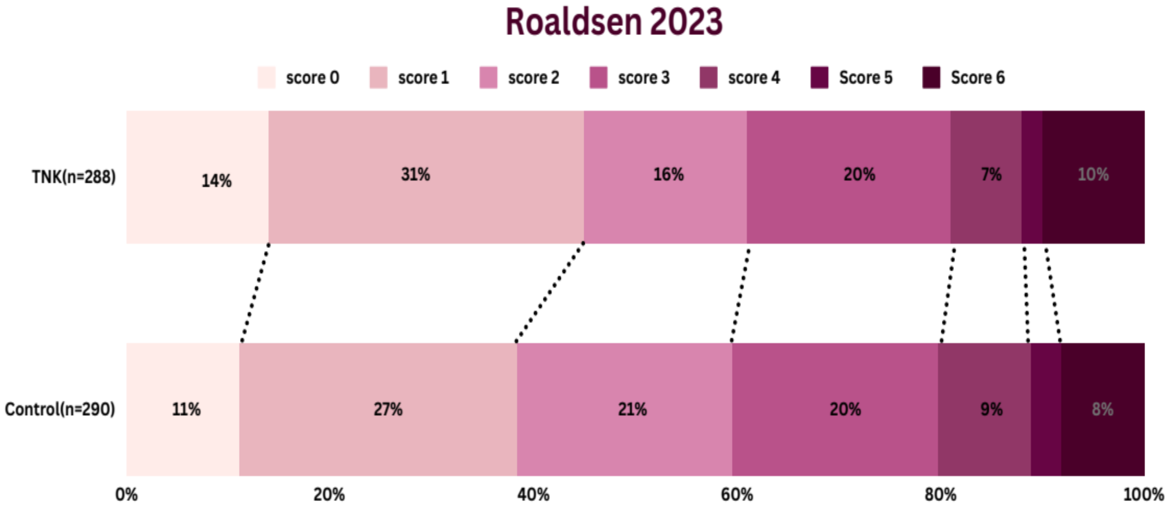

2. Wang 2023

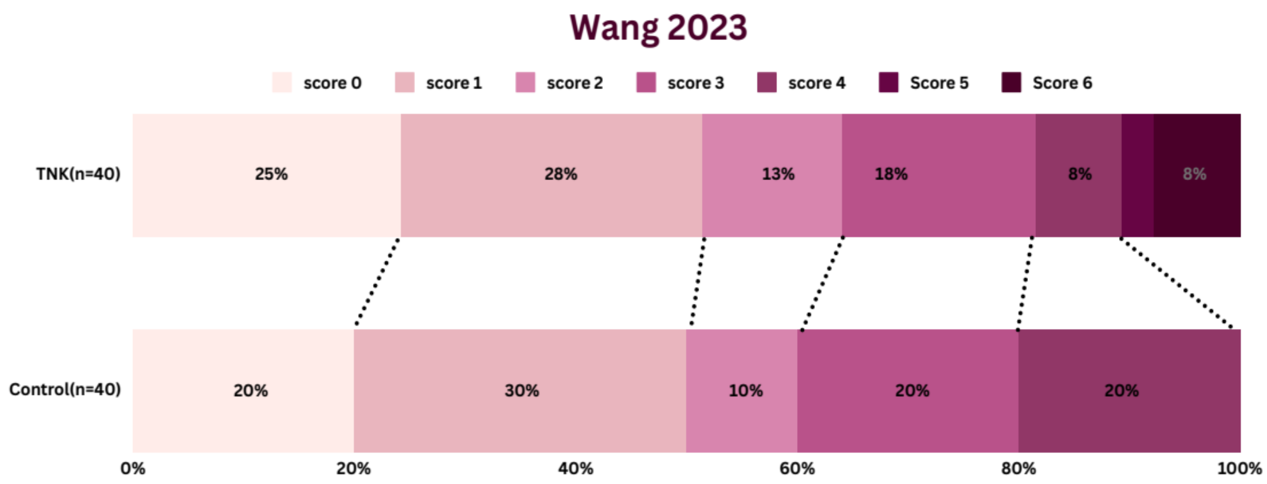

3. Albers 2024

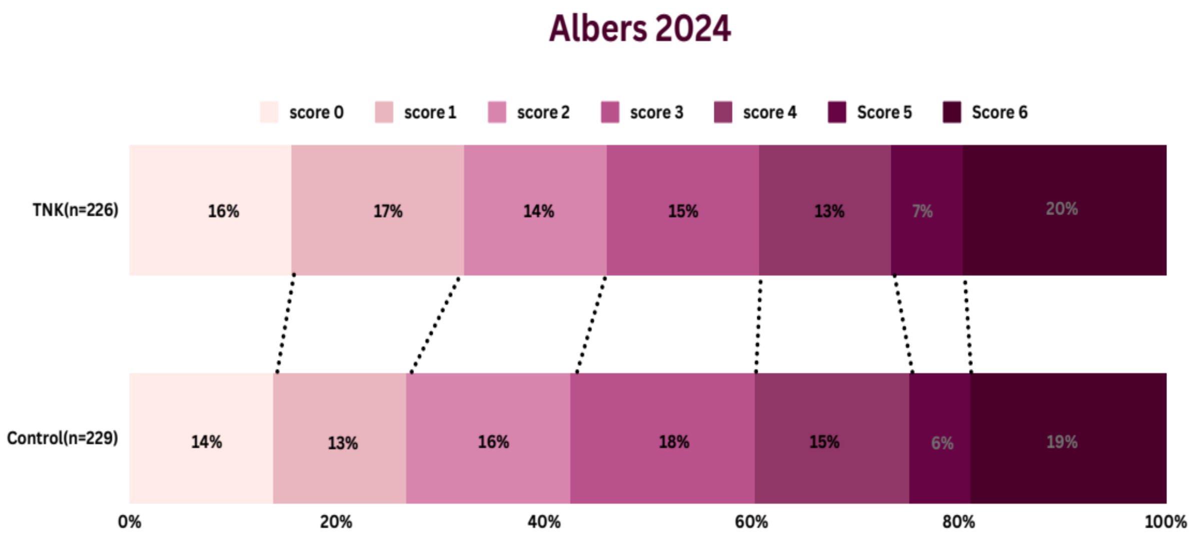

4. Cheng 2024

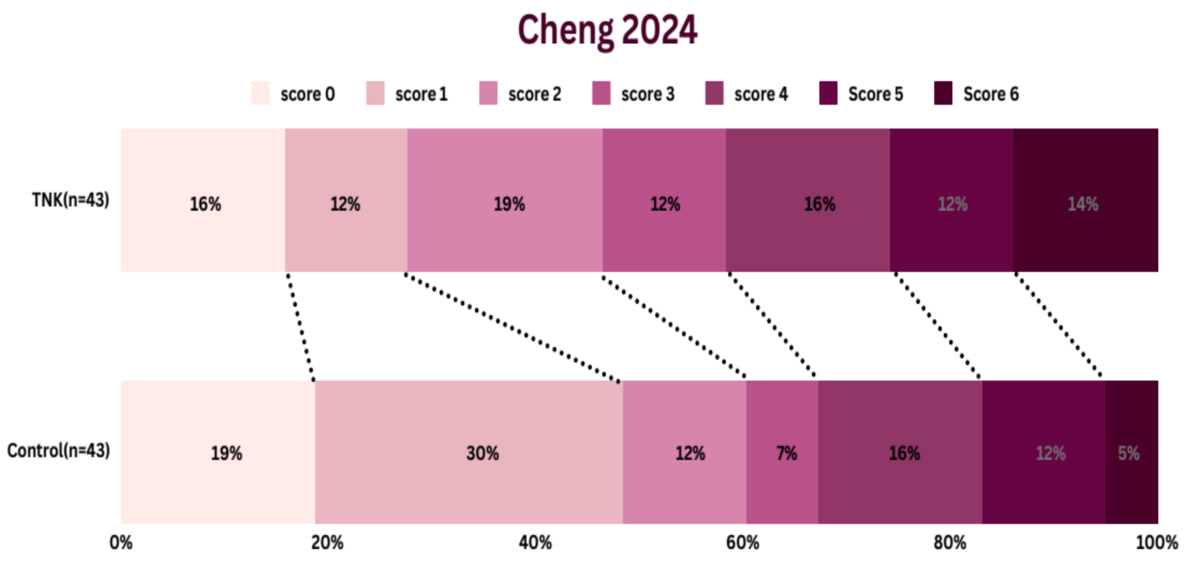

5. Coutts 2024

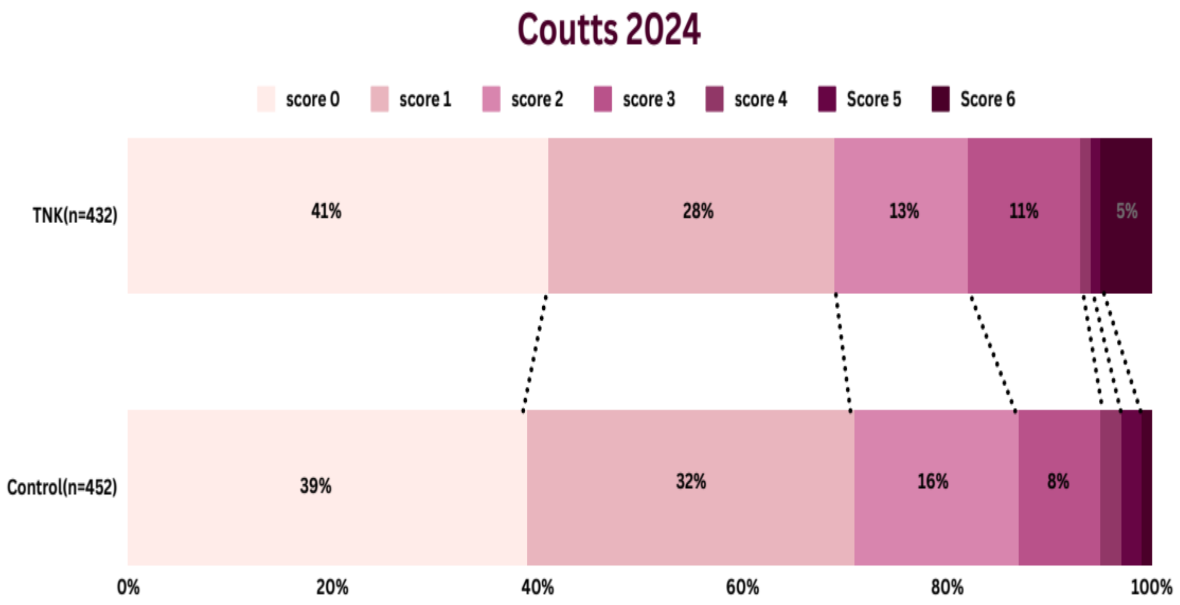

6. Xiong 2024

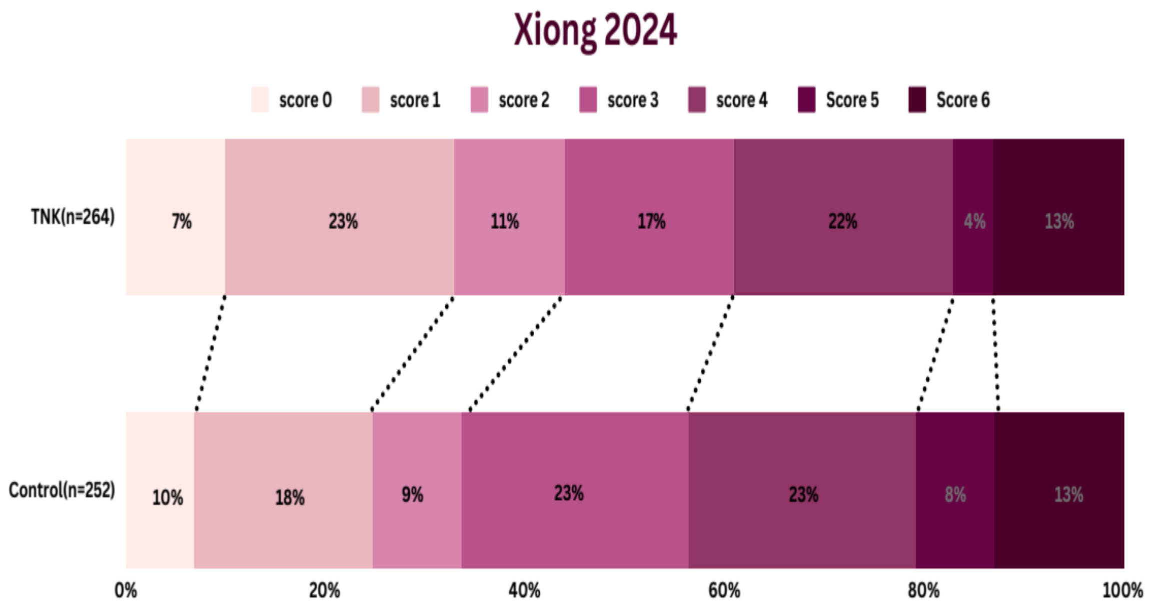
Xiong 2024
score 0
score 1
score 2
score 3
score 4
Score 5
Score 6
TNK(n=264)
7%
23%
11%
17%
22%
4%
13%
Control(n=252)
10%
18%
9%
23%
23%
8%
13%
0%
20%
40%
60%
80%
100%

7. Cheng 2025

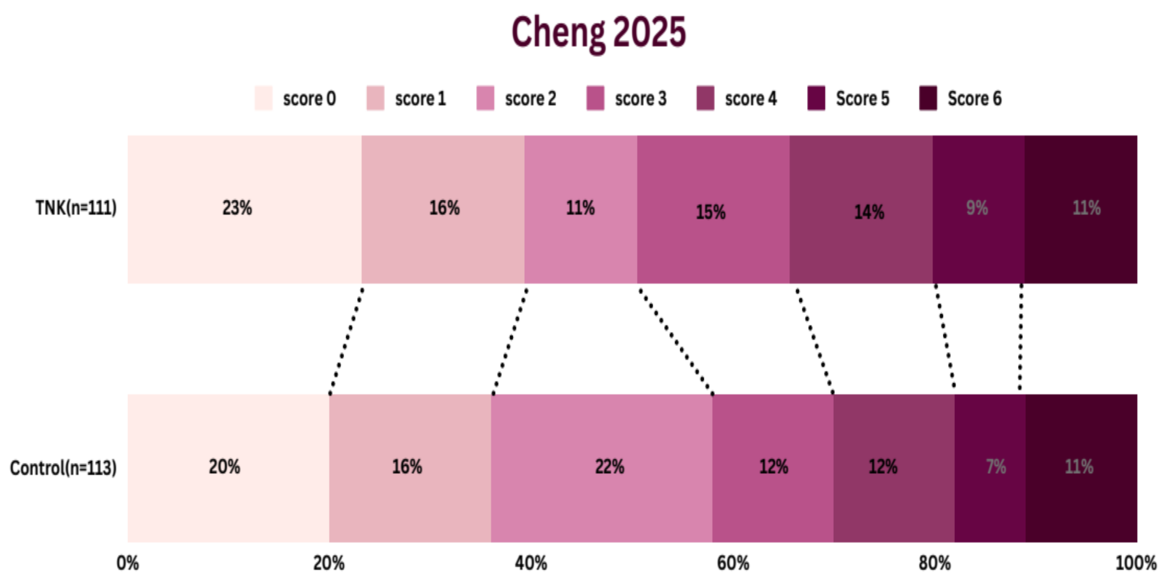
Cheng 2025
score 0
score 1
score 2
score 3
score 4
Score 5
Score 6
TNK(n=111)
23%
16%
11%
15%
14%
9%
11%
Control(n=113)
20%
16%
22%
12%
12%
7%
11%
0%
20%
40%
60%
80%
100%

8. Chen 2024

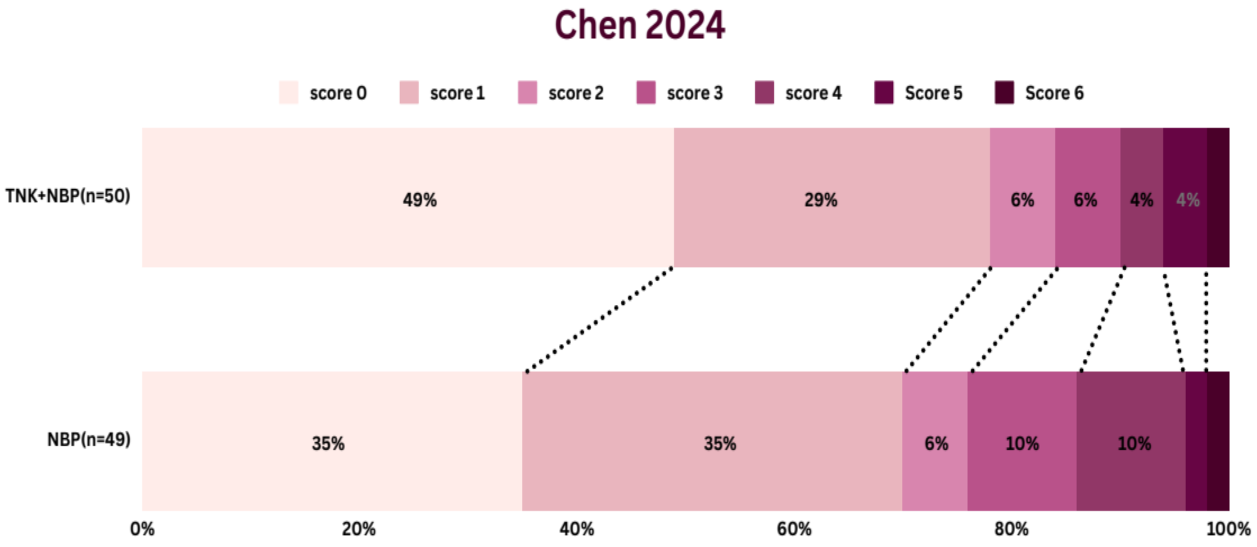

9. Yogendrakumar 2025

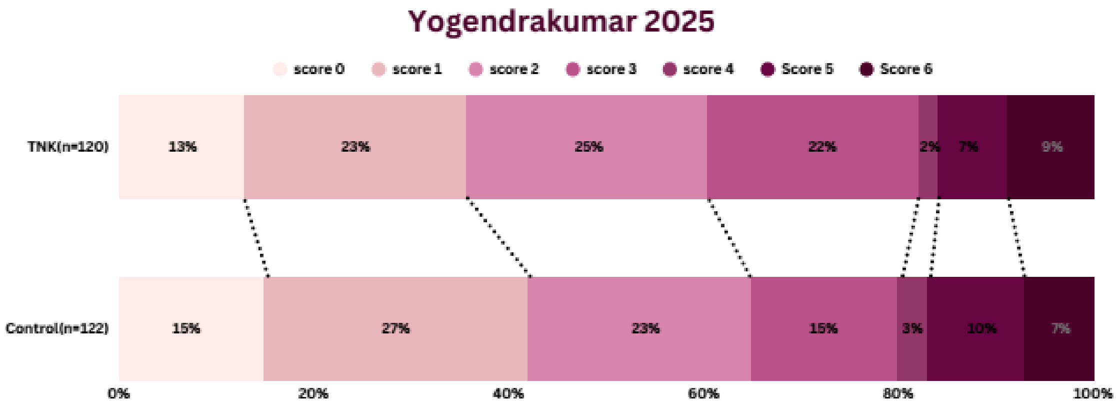

10. Overall distribution at 90 days

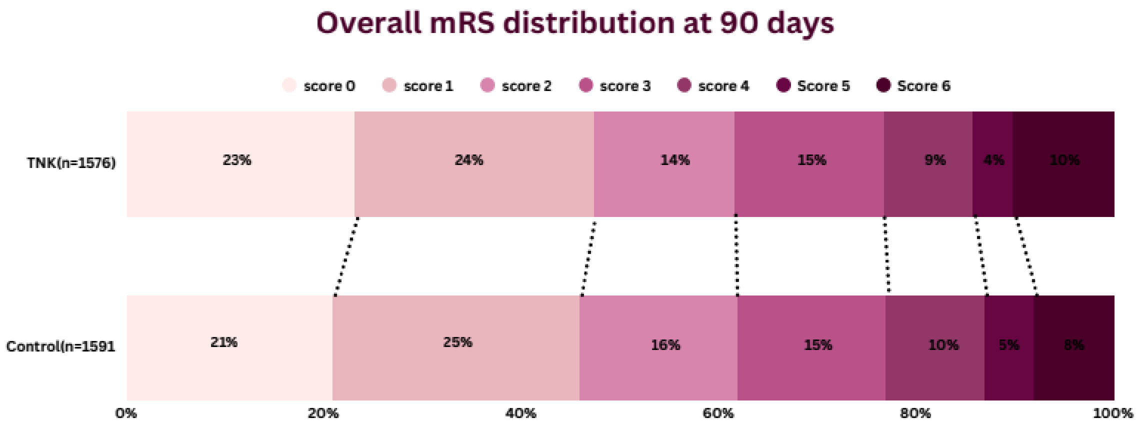

Quality assessment results

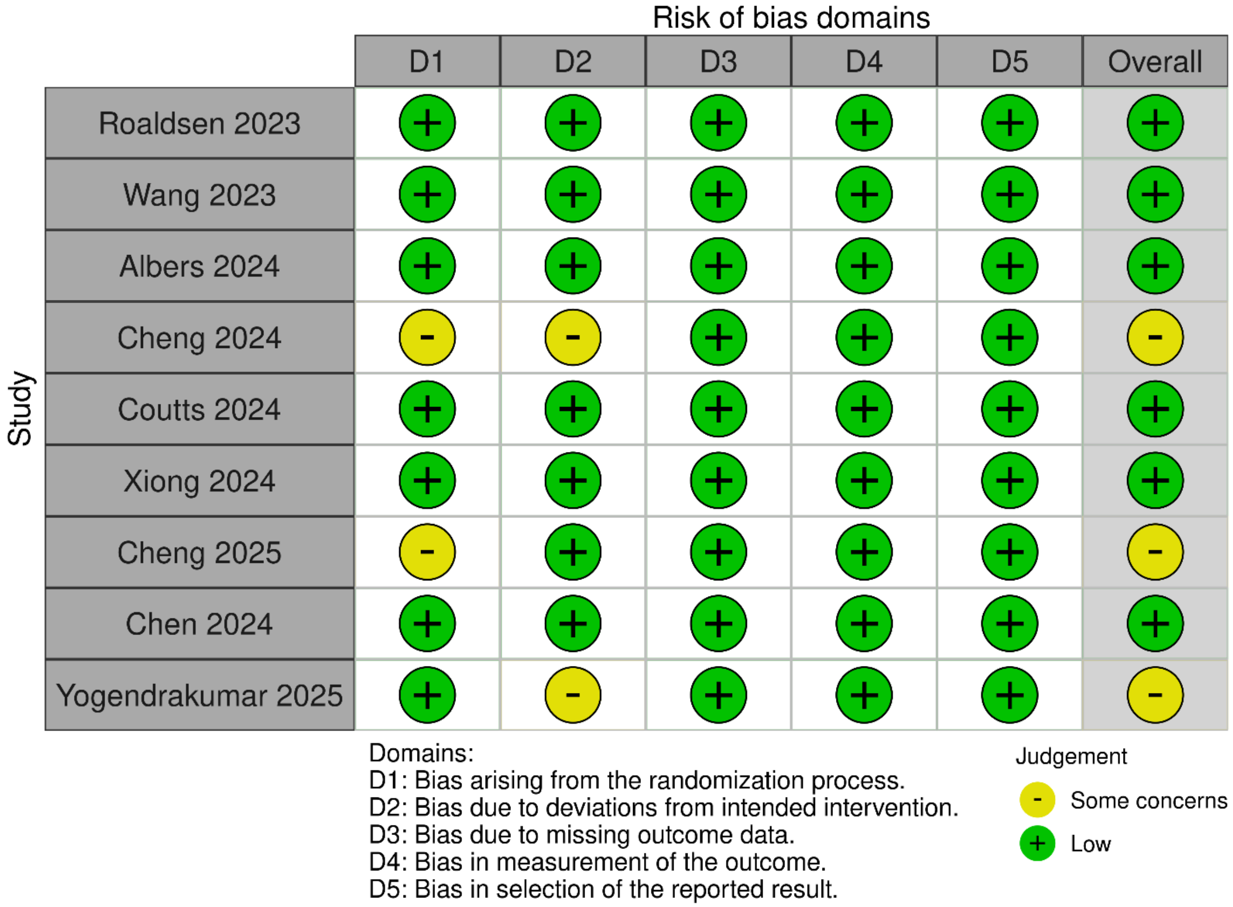
Risk of bias domains
D1
D2
D3
D4
D5
Overall
Study
Roaldsen 2023
Wang 2023
Albers 2024
Cheng 2024
Coutts 2024
Xiong 2024
Cheng 2025
Chen 2024
Yogendrakumar 2025
Domains:
D1: Bias arising from the randomization process.
D2: Bias due to deviations from intended intervention.
D3: Bias due to missing outcome data.
D4: Bias in measurement of the outcome.
D5: Bias in selection of the reported result.
Judgement
Some concerns
Low

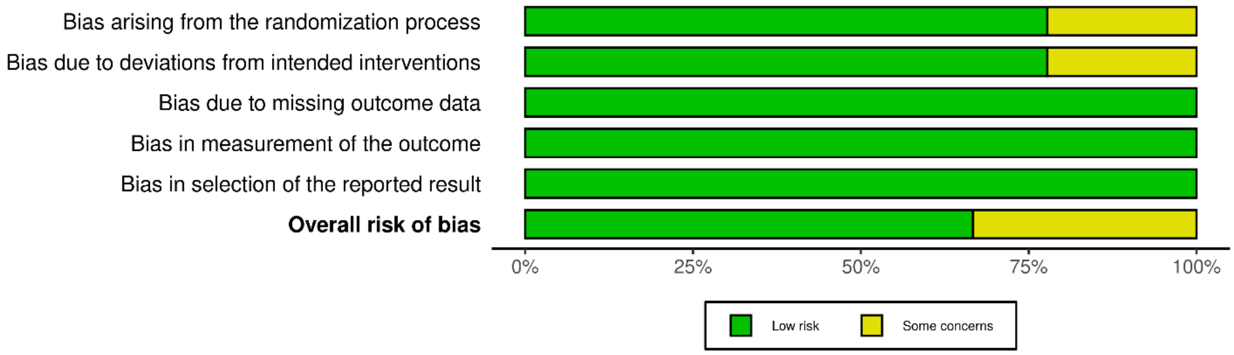
Bias arising from the randomization process
Bias due to deviations from intended interventions
Bias due to missing outcome data
Bias in measurement of the outcome
Bias in selection of the reported result
Overall risk of bias
0%
25%
50%
75%
100%
Low risk
Some concerns
